# Anti-neoplastic action of Cimetidine/Vitamin C on histamine and the PI3K/AKT/mTOR pathway in Ehrlich breast cancer

**DOI:** 10.1038/s41598-022-15551-6

**Published:** 2022-07-07

**Authors:** Sherihan Salaheldin Abdelhamid Ibrahim, Sarah A. Abd El-Aal, Ahmed M. Reda, Samar El Achy, Yasmine Shahine

**Affiliations:** 1 Department of Pharmacology and Therapeutics, Faculty of Pharmacy, Pharos University in Alexandria, Alexandria, Egypt; 2 Department of Pharmacy, Kut University College, Al Kut, Wasit, 52001 Iraq; 3 Department of Biochemistry, Faculty of Pharmacy, Egyptian Russian University, Badr City, Cairo Egypt; 4 Department of Pathology, Faculty of Medicine, Alexandria University, Alexandria, Egypt; 5 Department of Microbiology & Immunology, Faculty of Pharmacy, Pharos University in Alexandria, Alexandria, Egypt

**Keywords:** Biochemistry, Cancer, Immunology

## Abstract

The main focus of our study is to assess the anti-cancer activity of cimetidine and vitamin C via combating the tumor supportive role of mast cell mediators (histamine, VEGF, and TNF-α) within the tumor microenvironment and their effect on the protein kinase A(PKA)/insulin receptor substrate-1(IRS-1)/phosphatidylinositol-3-kinase (PI3K)/serine/threonine kinase-1 (AKT)/mammalian target of rapamycin (mTOR) cue in Ehrlich induced breast cancer in mice. In vitro study was carried out to evaluate the anti-proliferative activity and combination index (CI) of the combined drugs. Moreover, the Ehrlich model was induced in mice via subcutaneous injection of Ehrlich ascites carcinoma cells (EAC) in the mammary fat pad, and then they were left for 9 days to develop obvious solid breast tumor. The combination therapy possessed the best anti-proliferative effect, and a CI < 1 in the MCF7 cell line indicates a synergistic type of drug interaction. Regarding the in vivo study, the combination abated the elevation in the tumor volume, and serum tumor marker carcinoembryonic antigen (CEA) level. The serum vascular endothelial growth factor (VEGF) level and immunohistochemical staining for CD34 as markers of angiogenesis were mitigated. Additionally, it reverted the state of oxidative stress and inflammation. Meanwhile, it caused an increment in apoptosis, which prevents tumor survival. Furthermore, it tackled the elevated histamine and cyclic adenosine monophosphate (cAMP) levels, preventing the activation of the (PKA/IRS-1/PI3K/AKT/mTOR) cue. Finally, we concluded that the synergistic combination provided a promising anti-neoplastic effect via reducing the angiogenesis, oxidative stress, increasing apoptosis,as well as inhibiting the activation of PI3K/AKT/mTOR cue, and suggesting its use as a treatment option for breast cancer.

## Introduction

Breast cancer is one of the most common types of neoplasia that causes mortality and morbidity among women worldwide^1^. Despite the presence of effective conventional cancer treatments such as chemotherapy, radiation, and/or surgery, patients usually face many side effects from these treatments. There is an urgent need to find new treatment options for breast cancer to improve the quality of life of those patients.

Ehrlich-induced breast cancer is an aggressive undifferentiated mammary adenocarcinoma that develops in mice. It is a simple model that possesses a short duration for induction and resembles the pathogenesis of breast cancer in humans, so it is usually used to investigate new promising drugs in cancer^2^.

The tumor microenvironment (TME) has been extensively studied for the role of its resident cells, such as fibroblasts, endothelial cells, cancer stem cells, and immune cells, in the maintenance, recurrence, invasiveness, and treatment resistance of the cancer cells^3^. Paul Ehrlich was the first who reported mast cells' presence, which is also named tumor-associated mast cells (TAMCs), in different types of human solid tumors, especially breast cancer^4^. The role of TAMCs in the TME is still controversial. On the one hand, some researchers reported that mast cell infiltration predicts improved prognosis in some types of cancer^5^. On the other hand, TAMCs play an important role in the tumor progression via the production of pro-angiogenic factors such as histamine, vascular endothelial growth factor (VEGF), tumor necrosis factor-alpha (TNF-α), and fibroblast growth factor (FGF)^4^. The CD34 is considered a specific marker for tumor angiogenesis, as it can identify the condition of new blood vessel formation during tumor growth^6^.

Histamine is mainly produced from basophils and TAMC in the TME. The elevated level of histamine and the expression levels of histamine receptors (H1R-H4R) in the TME, play an important role in tumor progression. Moreover, histamine can interact with the HRs to recruit more and more TAMCs leading to a vicious cycle^7^.

Furthermore, there are many immune cells in the TME that can produce pro-inflammatory cytokines such as TNF-α, and reactive oxygen species (ROS) that subsequently lead to a state of inflammation and oxidative stress. Oxidative stress is characterized by shifting the balance towards oxidant systems (ROS and Malondialdehyde (MDA)) more than antioxidant systems (superoxide dismutase (SOD), and reduced glutathione (GSH)). These factors act together to alter the proteins responsible for DNA repair and apoptosis, favoring tumor survival, proliferation, migration, and metastasis^4,8^.

Moreover, activation of the PI3K/AKT/mTOR cue plays a role in angiogenesis, resistance to apoptosis, and survival of breast tumor cells. This pathway can be activated directly via elevated levels of ROS and VEGF in the TME^9^. Also, the histamine released from the TAMC can indirectly activate the PI3K/AKT/mTOR pathway. This can be explained as follows: the histamine from TAMC acts on H_2_R, and an increase in the level of cyclic adenosine monophosphate (cAMP) followed by protein kinase A (PKA) occurs^10^. Furthermore, the PKA can modulate the phosphorylation of Insulin Receptor Substrate 1 (IRS1), to activate PI3K/AKT/mTOR pathway^11^.

Apoptosis is a programmed cell death that is involved in several physiological and pathological conditions. During carcinogenesis, evasion of apoptosis occurs via an imbalance between pro-apoptotic and anti-apoptotic proteins, as well as impairment in the caspase-3 enzyme. This leads to prolonged survival of tumor cells^12^.

Cimetidine (CIM) is an antagonist for the H_2_R, and it is mainly used in the treatment of gastrointestinal ulcers due to its ability to reduce gastric acid secretion. It possesses an anti-tumor activity against various types of cancers, including colon, gastric, kidney cancers, and melanomas, by preventing histamine from binding to H_2_R, inhibiting tumor growth, and antagonizing selectins' action, which reduces cancer metastasis^13,14^.

Vitamin C, also known as ascorbic acid, is a water-soluble vitamin that is essential for collagen, catecholamine, and carnitine biosynthesis. Also, it acts as a cofactor for several enzymes and an antioxidant to protect cells from damage by oxidative stress^15^. Furthermore, it has been shown that vitamin C can be used for the treatment of cancer as well as other pathological conditions^15,16^. Its anti-neoplastic activity is mediated via inhibition of the tumor growth, and by inducing apoptosis. Additionally, numerous studies have found vitamin C inversely correlated with histamine. It can act as a mast cell stabilizer, prevent the formation of histamine via inhibition of histidine decarboxylase, and increase the breakdown of histamine via activation of diamine oxidase^15^.

The main objective of this study is to assess the anti-tumor activity of cimetidine and vitamin C via mitigating the tumor-associated mast cell mediators in the TME and inhibiting the activation of the PI3K/AKT/mTOR trajectory.

## Materials and methods

### In vitro experiment

#### Cell line

The MCF-7 human breast adenocarcinoma cells (86,012,803) were purchased from (Sigma Aldrich, USA). The cells were grown in EMEM (EBSS) (Gibco) + 2 mM Glutamine (Gibco) + 1% non-essential amino acids (NEAA)(Gibco) + 10% fetal Bovine Serum (FBS)/FCS (Gibco) in 75 cm^2^ culture flask. The cells were incubated at 37 °C and 5% CO_2_ in an air atmosphere until tested the cells.

#### MTT cell proliferation assay

Each well was seeded with (1 × 10^4^) cells in ninety-six-well culture plates and incubated for 24 h at 37 °C. Then, cells were treated with serial two-fold dilutions of various concentrations of each of the following drugs: cimetidine, vitamin C, and a combination of cimetidine/vitamin C. Three wells were used for each concentration of the tested drugs, and the incubation was continued for 48 h. Then, 20 μL of 5 mg/mL MTT (Invitrogen) was added to each well and incubated for 4 h. At 37 °C in the culture hood, MTT was replaced by 150 μL of DMSO. The plate was covered with tinfoil and agitated on an orbital shaker for 15 min. The optical density was measured at 490 nm using a microplate reader (Biotek ELX-800, USA). The following equation was used: % of cell viability = At Ac × 100, where (At) is the absorbance of the drug, and (Ac) is the absorbance of the control^17^.

The CompuSyn software (version 1.0.1) was used to assess the combination index (CI) of cimetidine with vitamin C in vitro, which reveals the type of pharmacologic drug interactions. Values of the CI < 1 reflect synergistic interactions, whereas CI = 1 indicates additive effects. Drug antagonism is indicated by CI values > 1^18^.

### In vivo experiments

#### Animals

Female Swiss albino mice (6–7 weeks, 20–25 gm weights) were purchased from Pharos University in Alexandria and kept under observation for one week prior to the study, with free access to food and water. All experimental procedures were performed in strict accordance with the guide for the Care and Use of Laboratory Animals (NIH), the Animal Research: Reporting of In Vivo Experiments (ARRIVE) guidelines, and approved by the "Unit of research Ethics Approval committee, Pharos University in Alexandria" (PUA-0120219263029). Animal and biological wastes were safely disposed at the end of the study.

#### Ehrlich induced breast solid tumor

Mice with Ehrlich ascites carcinoma (EAC) were purchased from the Egyptian National Cancer Institute (NCI; Cairo University, Egypt). Ascites solution was withdrawn from the peritoneal cavity of mice, and the count of tumor cells in the ascites fluid was assessed via a Neubauer Hemocytometer. Moreover, the cell viability was determined according to the previously described method^19^. Then, Ehrlich ascites carcinoma cells (EAC) were suspended in phosphate buffer saline (PBS). Each mouse in the experimental group received 0.2 mL EAC saline solution (2.5 × 10^6^ cells) subcutaneously in the mammary fat pad on day 0. Mice were left for 9 days with free access to water and food to develop solid breast cancer^20^.

#### Experimental animal groups

Every 10 mice were allocated into a group forming 5 groups: Group (1): involved healthy mice (the negative control group); Group (2): involved the untreated Ehrlich induced solid breast cancer mice treated with both solvents of the used drugs (the positive control group). Group (3): involved mice treated with cimetidine dissolved in 0.5% carboxymethyl cellulose sodium and injected peritumoral at a dose of (200 mg/kg/day)^4^; Group (4): involved mice treated with vitamin C, dissolved in water and injected peritumoral at a dose of 4gm/kg/day^8^; Group (5): involved mice treated with both cimetidine and vitamin C in the aforementioned doses. Tested drugs were administered daily for 15 days, from the 10th day till the end of the study.

### Assessment of Ehrlich induced solid tumor volume

We assessed the tumor volume (V) every other day until the last record on the 25^th^-day post-implantation, just before scarifying the survived mice. Using a vernier caliper, tumor volume was calculated using the following formula: V = (length + [width]^2^)/2^21^.

### Serum and tumor tissue collection

Collection of serum and breast cancer tissue samples was carried out at the end of the study. On the 25th day, mice were anesthetized via ketamine and xylene. Then, blood samples were collected from the orbital sinus in non-heparinized glass tubes and left for 30 min at room temperature to clot. The samples were then centrifuged at 5000 rpm for 10 min to obtain the serum that was stored at -80 °C. All mice were sacrificed by cervical dislocation, and the tumor was excised from each mouse, washed immediately with ice-cold saline, and blotted dry on filter paper. Finally, the tumor sample was divided into two parts. The first part was preserved in 10% formaldehyde solution overnight and processed for the formation of paraffin-embedded blocks to be used in histopathological and immunohistochemical assessments. The second part was preserved at − 80 °C for the determination of different parameters on the biological and molecular levels.

### Serum parameters

#### Determination of serum levels of carcinoembryonic antigen (CEA) and VEGF

The serum levels of CEA (Cat no. E4741, Biovision, Northern California), and VEGF (Cat no. BMS619-2, Thermofisher Scientific**,** USA**),** were determined by using enzyme-linked immune sorbent assay (ELISA) kits according to the manufacturer's instructions.

### Tissue parameters

#### Homogenization

Normal mammary gland tissues and tumor tissues were homogenized with ice-cold 1.15% KCl and 0.01 mol/L sodium–potassium phosphate buffer (pH 7.4), followed by centrifugation at 4 °C for 20 min at 10,000×*g*.

#### Determination of oxidative stress parameters

The obtained supernatant from the homogenization process was used to determine the tumor tissue levels of the following oxidative stress parameters, GSH (Cat no. MBS026635, My BioSource, USA), MDA (Cat no. MBS741034, My BioSource, USA), and SOD (Cat no. MBS034842, My BioSource, USA) by using colorimetric assay kits according to the manufacturer's instructions.

#### Determination of inflammatory parameter (TNF-α)

The TNF-α levels in the normal mammary gland tissues as well as tumor tissues were estimated by ELISA kit according to the manufacturer's instructions (Cat no. MBS825075, My BioSource, USA).

#### Determination of histamine level

According to the manufacturer's instructions, histamine concentration in the mammary gland and tumor microenvironments was determined using a competitive, colorimetric ELISA kit (Cat no MBS005252, My BioSource, USA).

#### Determination of cAMP level

The intracellular cAMP level was measured by a colorimetric competitive ELISA kit (Cat no ab65355, Abcam, USA) according to the manufacturer's instructions.

### Reverse transcription-polymerase chain reaction (RT-PCR)

Total RNA was extracted from breast cancer and normal mammary tissues homogenates by using RNAiso Plus (Takara Bio, Inc.), according to the manufacturer's protocol. Then, the RNA samples were reverse transcribed to the cDNA at 37 °C for 15 min subsequently amplified as follows (35 cycles: 30 s at 94 °C, 30 s at 55 °C, and 60 s at 72 °C), using the Superscript One-Step RT-PCR System (Cat no.12594100, Invitrogen, USA) with gene-specific oligonucleotide primers (Table 1). The Stratagene MX3005P software determined amplification curves and Ct values. The primer efficiencies for all primer pairs used were 98%. To estimate the variation of gene expression on the RNA of the different samples, the Ct of each sample was compared with that of the positive control group. Relative IRS-1 mRNA expression level was calculated using the "2-ΔΔCt" method.

**Table 1:** The primers sequences used were as follows:

Gene	Forward sequence	Reverse sequence
IRS1	5′-TGTCACCCAGTGGTAGTTGCTC-3′	5′-CTCTCAACAGGAGGTTTGGCATG-3′
GAPDH	5′-AGGTCGGTGTGAACGGATTTG-3′	5′-TGTAGACCATGTAGTTGAGGTCA-3′

### Western blot technique to determine the PKA-C α/β, total and phosphorylated PI3K, AKT, mTOR expression levels in the normal mammary fat pad as well as in Ehrlich induced breast tumor

Fixed paraffin-embedded tissue samples were deparaffinized, hydrated in xylene, descending alcohol solutions, and rinsed with PBS. Proteins were extracted using Qproteome FFPE Tissue Kit (Cat no. 37623, Qiagen, Italy) by incubation in an extraction buffer at 100 °C for 20 min, then incubated for 2 h at 80 °C according to the manufacturer's manual. The sample was then cooled to 4 °C for 5 min and centrifuged (14,000×*g*, 15 min, 4 °C). The supernatant was transferred to a new collection tube. Afterward, extracted lysates were resolved in 10% polyacrylamide SDS-PAGE gel in a BioRad Mini Protean tetra cell system at 150 V for 1 h. (Bio-Rad protein assay kit) according to the manufacturer's instructions. Electrophoresed proteins were transferred into a nitrocellulose membrane, and then the membranes were blocked at room temperature in TBST (100 mM Tris pH 7.5, 0.9% NaCl, 0.1% Tween 20) plus 5% non-fat dry milk for 1 h and incubated overnight at 4 °C with the primary antibodies. The primary antibodies were as follows, PKA-C α/β antibody (Cat no. MAB5908, R and D systems, Minneapolis, MN, USA), PI3K (Cat. no. ab191606; Abcam), Phospho-PI3K p85 alpha (Tyr607) Polyclonal Antibody (Cat no. PA5-38905, Thermofisher Scientific, USA), AKT (Cat no. 4685; Cell Signaling Technology, Inc.), Phospho-AKT1 (Ser473) Monoclonal Antibody (Cat no. 44-621G, Thermofisher Scientific, USA), mTOR (Cat no. 2983; Cell Signaling Technology, Inc.) and Phospho-mTOR (Ser2454) Polyclonal Antibody (Cat no. PA5-105571, Thermofisher Scientific, USA). Then, the sections were washed three times with TBST and incubated with goat anti-rabbit-HRP secondary antibody (Cat no. # 31460, Thermofisher Scientific, USA) at a dilution of 1:20,000 and finally, immunolabeled proteins were detected by incubation with ECL substrate and the chemiluminescent signals were captured using a CCD camera-based imager. Image analysis software was used to read the band intensity of the target proteins against the control sample after normalization by beta-actin on the Chemi Doc MP imager^22^.

### Histopathological and histomorphometric assessment

Five μm slices from tumor blocks were sectioned and stained with hematoxylin–eosin (H&E) for histopathological examination, mitotic counts in 10 high power fields (HPF), and morphometric assessment of the area of tumor necrosis using the Leica Application Suite, Version 4.12.0 (Leica Microsystems CMS GmbH) image analysis program.

### Immunohistochemistry

As previously described, tissue-embedded paraffin blocks were sectioned on positively charged slides, deparaffinized, and rehydrated. Citrate buffer (pH 6.0) was used for heat-induced antigen retrieval. Endogenous peroxidase was blocked with H_2_O_2_ and endogenous biotin with the aid of a blocking buffer (Cat no 32052, Thermofisher Scientific, USA). The sections were treated with primary antibodies against Caspase-3 (Cat no 9662, Cell Signaling Technology, MA, USA) with a dilution of (1:1000), and CD34 (Cat no 3569, Cell Signaling Technology, MA, USA) with a dilution of (1:1000). Immunoreactions were developed using the Ultra-sensitive ABC Peroxidase Mouse IgG Staining Kit (Cat no 32052, Thermofisher Scientific, USA) using the biotinylated secondary antibody. DAB (Cat no 34065, Thermofisher Scientific, USA) was used as chromogen, and the tissue was counterstained with Mayer's Hematoxylin.

Caspase-3 stained sections were assessed by counting the number of positively stained cells (cytoplasmic) in 10 HPFs at the invasive front of the tumor, away from the central areas of necrosis. CD34 stained sections were assessed by counting the number of positively stained endothelial cells lining vascular spaces within the tumor in 10HPFs. All assessments were conducted using the Leica Application Suite, Version 4.12.0 (Leica Microsystems CMS GmbH) image analysis software.

### Data analysis

All statistical analyses were conducted using GraphPad Prism v. 5.03 software (GraphPad Software, San Diego, CA, USA). Data were expressed as mean ± SD (n = 8). Statistical differences between groups were determined using one-way ANOVA, followed by Tukey's post hoc multiple comparison test. A p-value less than 0.05 was considered significant. The CompuSyn software (version 1.0.1) was used to estimate the synergistic effects of different drug combinations by generating the combination index (CI), where CI < 1, CI = 1, and CI > 1 designated synergism, additive effect, and antagonism, respectively. Using the Spearman coefficient test, statistical correlations between different parameters were performed by IBM SPSS software package version 20.0 (Armonk, NY: IBM Corp). A p-value less than 0.05 was considered significant.

### Ethical approval

Experimental protocol and procedures were complied with the regulations declared by the ARRIVE (Animal Research: Reporting of In Vivo Experiments) guidelines. The protocol was approved by the "Unit of research Ethics Approval committee, Pharos University in Alexandria" (PUA01202109263029).

## Results

### In vitro study

#### MTT assay using MCF-7 human cell line

**Figure 1:**
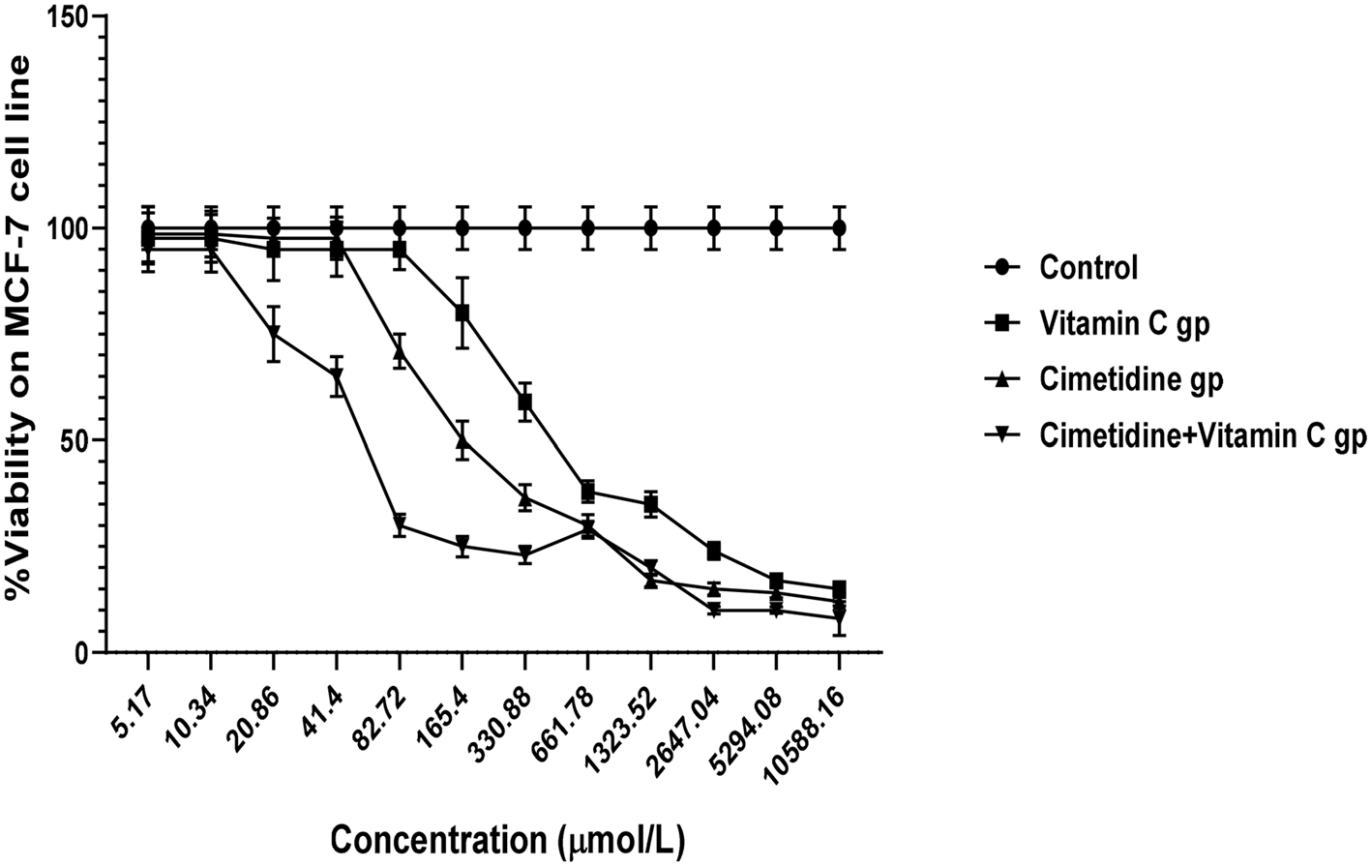
Effects of serial two-fold dilutions of vitamin C, cimetidine, and their combination on % of cell viability of MCF7 cells using MTT assay.

The IC50 values of vitamin C and cimetidine after being added to the culture medium of the MCF7 cell line were (373.73 and 144.17 μmol/L, respectively) (Table 2, Fig. 1, and Supplementary 6). On the other hand, adding a combination of vitamin C/cimetidine to the culture medium of the MCF7 cell line resulted in a 3.42-fold reduction of IC50 (42.16 μmol/L) compared to the cimetidine treated medium.

**Table 2:** Evaluation of the half-maximal inhibitory concentrations (IC50) of tested drugs on MCF-7 human cell line using MTT assay.

Treatment	IC50 (μmol/L) (MCF-7)
Vitamin C	373.73 ± 28.54
Cimetidine	144.17 ± 12.34
Cimetidine + Vitamin C	42.16^$^ ± 2.56

Data are presented as the mean ± SD of the IC50 (μ mol/L), n = 3. $ is significantly different from cimetidine at p-value < 0.05.

#### In vitro determination of the combination index (CI)

**Figure 2:**
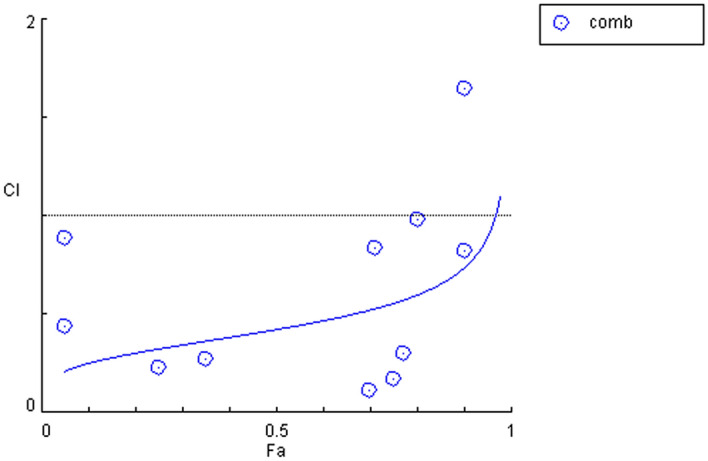
Combination index plot of vitamin C combined with cimetidine with serial two-fold dilutions at a constant ratio (1:1) in the MCF7 human cell line. Values of the CI < 1 reflect synergistic interactions.

The CI analysis using CompuSyn software (version 1.0.1) of different concentrations of cimetidine combined with vitamin C on the MCF-7 human cell line showed a synergistic effect (CI < 1) (Table 3 and Fig. 2).

**Table 3:** Combination Index analysis using different cimetidine and vitamin C concentrations at a constant ratio (1:1).

Cimetidine + Vitamin C (μmol/L)	Fa	CI
10.34	0.05	0.43977
20.86	0.05	0.88719
41.4	0.25	0.22971
82.72	0.35	0.27152
165.4	0.7	0.11020
330.88	0.75	0.16798

*Fa* fraction affected and *CI* combination index.

### In vivo experiment

#### Assessment of Ehrlich induced solid tumor volume

**Figure 3:**
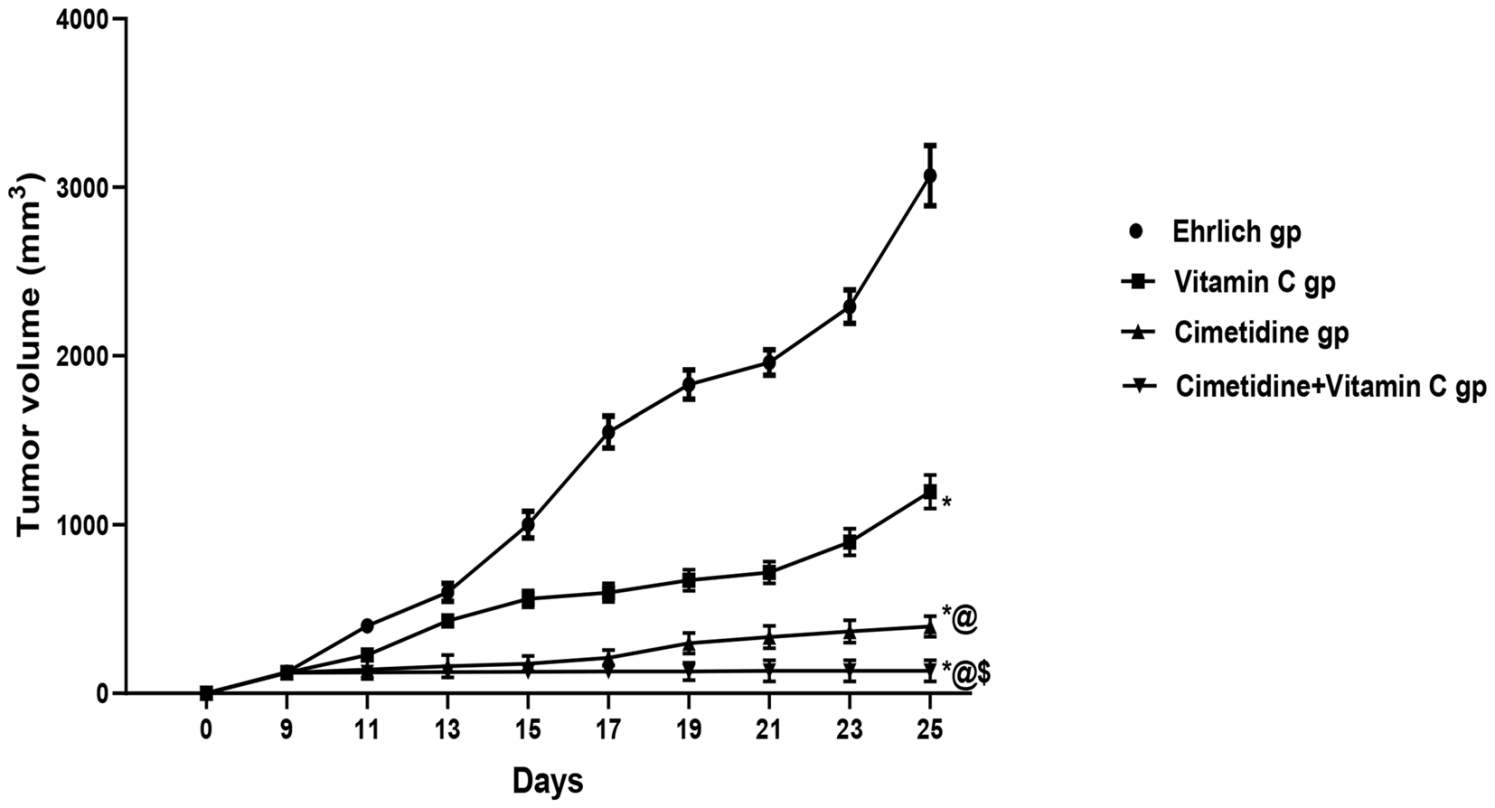
The changes in the tumor volume of Ehrlich induced solid breast cancer in mice following treatment with cimetidine at a dose of 200 mg/kg/day, vitamin C at a dose of 4 g/kg/day, and their combination for 15 days. Each point represents the mean ± SD of 10 mice. * is significantly different from Ehrlich untreated group, P < 0.05. @ is significantly different from the vitamin C-treated group, P < 0.05. $ is significantly different from the cimetidine-treated group, P < 0.05.

The effects of drug treatment on breast cancer growth are shown in (Table 4, Fig. 3, and Supplementary 6). Breast cancer growth was assessed by measuring tumor volume with a Vernier caliper from day 9 till the end of the study. All untreated mice showed an observable solid tumor in the mammary fat pad on day 9. Accordingly, tested drugs were administered on day 10. In the untreated mice, the tumor volume continued to increase progressively till the end of the treatment period. Cimetidine, vitamin C, and their combination significantly reverted tumor volume progression compared to the untreated group, on all of the follow-up days. On the 25th day, cimetidine caused more significant reduction in the tumor volume than that caused by vitamin C, P < 0.05. However, the combination of both drugs caused the most significant mitigation in the tumor volume compared to either cimetidine or vitamin C alone, P < 0.05.

**Table 4:** Effect of Cimetidine, Vitamin C, and their combination on tumor volume estimated by Vernier caliper in the mammary fat pad of mice in different groups.

Tumor volume (mm^3^)	Day 9	Day 11	Day 13	Day 15	Day 17	Day 19	Day 21	Day 23	Day 25
Ehrlich GP	126.13 ± 0.02	400.00 ± 0.04	600.00 ± 0.12	1000.00 ± 0.12	1550.00 ± 0.20	``1830.00 ± 0.10	1961.00 ± 0.10	2291.3 ± 0.12	3068.3 ± 0.14
Vitamin C GP	122.10 ± 0.18	227.98 ± 0.22*	429.65 ± 0.26*	559.89 ± 0.31*	597.32 ± 0.17*	670.79 ± 0.10*	716.50 ± 0.10*	897.00 ± 0.17*	1196.50 ± 0.10*
Cimetidine GP	121.98 ± 0.12	140.29 ± 0.02*@	160.50 ± 0.10*@	176.09 ± 0.20*@	210.45 ± 0.24*@	297.62 ± 0.10*@	335.00 ± 0.10*@	368.00 ± 0.16*@	397.00 ± 0.15*@
Cimetidine + Vitamin C GP	123.09 ± 0.08	125.00 ± 0.04*@	126.50 ± 0.08*@	128.61 ± 0.26*@	129.75 ± 0.42*@$	130.50 ± 0.10*@$	132.7 ± 0.10*@$	133.7 ± 0.02*@$	133.5 ± 0.12*@$

Significantly different from untreated group, *P < 0.05.

Significantly different from vitamin C-treated group, ^@^P < 0.05.

Significantly different from cimetidine-treated group, ^$^P < 0.05.

^@^Data are presented as mean ± SD.

#### Serum parameters

##### Serum CEA concentration

**Figure 4:**
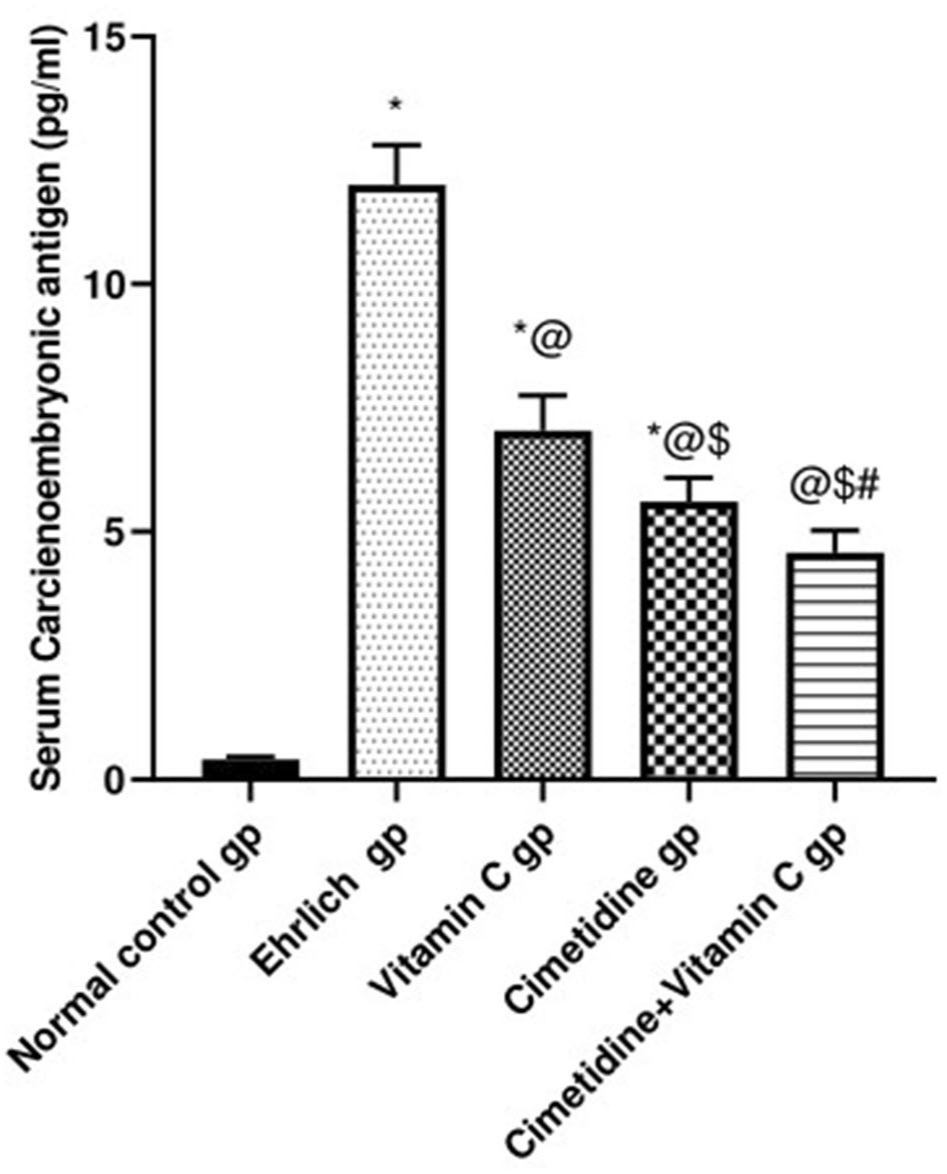
Changes in the serum CEA concentration following treatment with cimetidine at a dose of 200 mg/kg/day, vitamin C at a dose of 4 g/kg/day, and their combination for 15 days after Ehrlich induction of breast cancer in mice. Data are presented as mean ± SD.* Significantly different from the normal control group, P < 0.05. @ is significantly different from Ehrlich untreated group, P < 0.05. $ is significantly different from the vitamin C- treated group, P < 0.05. # is significantly different from the cimetidine-treated group, P < 0.05.

The CEA is a tumor marker, in which the changes in its serum level in different groups are illustrated in (Fig. 4, and Supplementary 6). Our results showed a significant provoking in the serum level of CEA in the Ehrlich induced breast cancer group (12 ± 0.80 pg/mL) compared to normal mice (0.4 ± 0.05 pg/mL), respectively. Cimetidine/vitamin C treatments at doses of 200 mg/kg/day and 4 g/kg/day mitigated the elevated serum level of CEA (5.52 ± 0.48 pg/mL) and (7 ± 0.71 pg/mL), respectively. The combination therapy caused the most significant averting in the elevated serum level of the aforementioned parameter (4.6 ± 0.44 pg/mL).

##### Serum VEGF concentration

**Figure 5:**
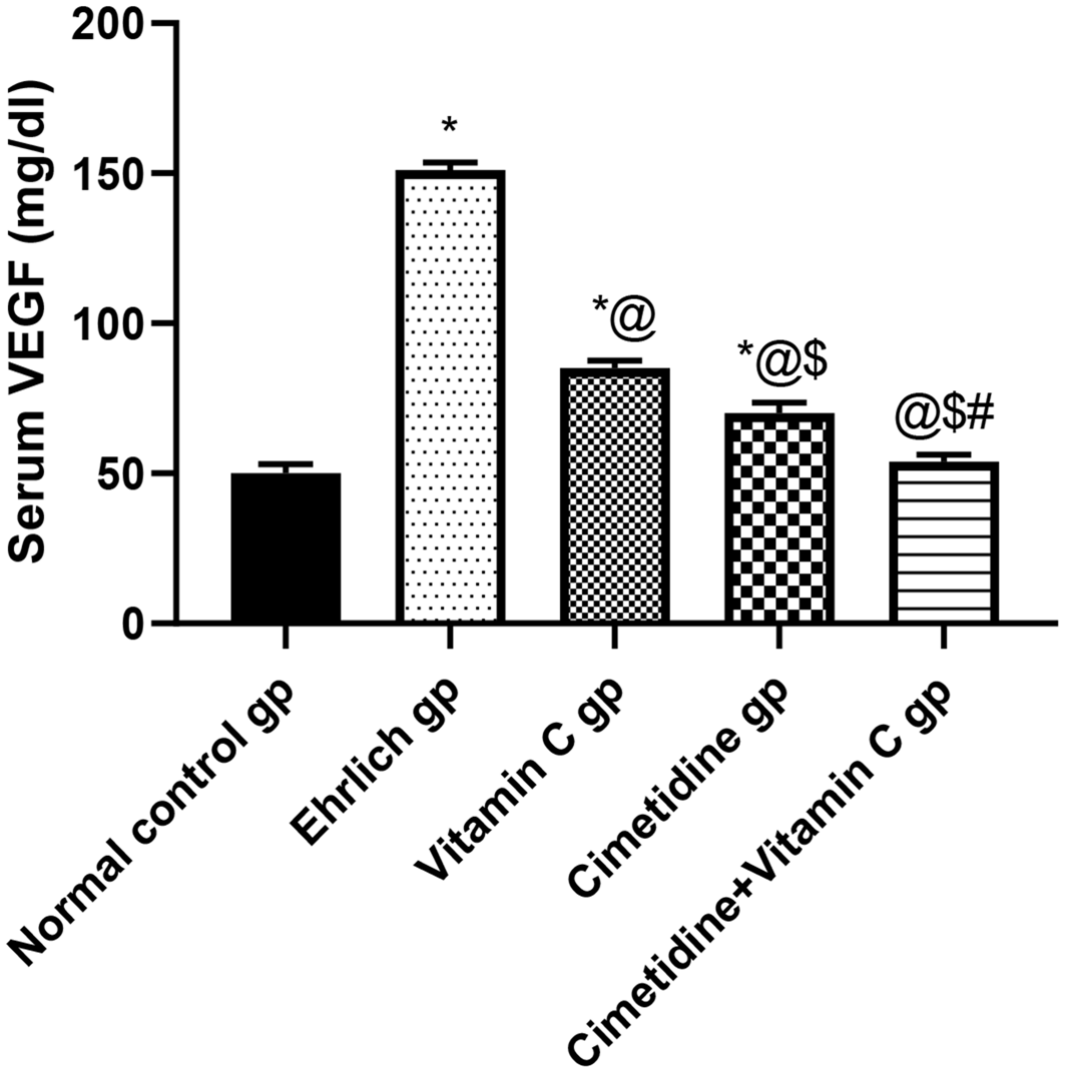
The changes in the serum VEGF concentration following treatment with cimetidine at a dose of 200 mg/kg/day, vitamin C at a dose of 4 g/kg/day, and their combination for 15 days after Ehrlich induction of breast cancer in mice. Data are presented as mean ± SD. * is significantly different from the normal control group, P < 0.05. @ is significantly different from Ehrlich untreated group, P < 0.05. $ is significantly different from the vitamin C- treated group, P < 0.05. # is significantly different from the cimetidine-treated group, P < 0.05.

The effect of treatment with cimetidine, vitamin C, and their combination on serum VEGF is presented in (Fig. 5, and Supplementary 6). There was 3.02 fold significant elevation in the serum level of VEGF in Ehrlich breast cancer mice compared to normal control mice. Cimetidine and vitamin C significantly suppressed the elevated serum VEGF levels by 53.64% and 43.71%, respectively, compared to untreated mice. The combination therapy caused the most significant decrease in the serum VEGF level by 64.3% compared to untreated Ehrlich-induced breast cancer-bearing mice. This highlighted the promising anti-angiogenic effects of the used drugs, with special emphasis on combination therapy.

#### Tissue parameters

##### Tissue levels of oxidative stress parameters

**Figure 6:**
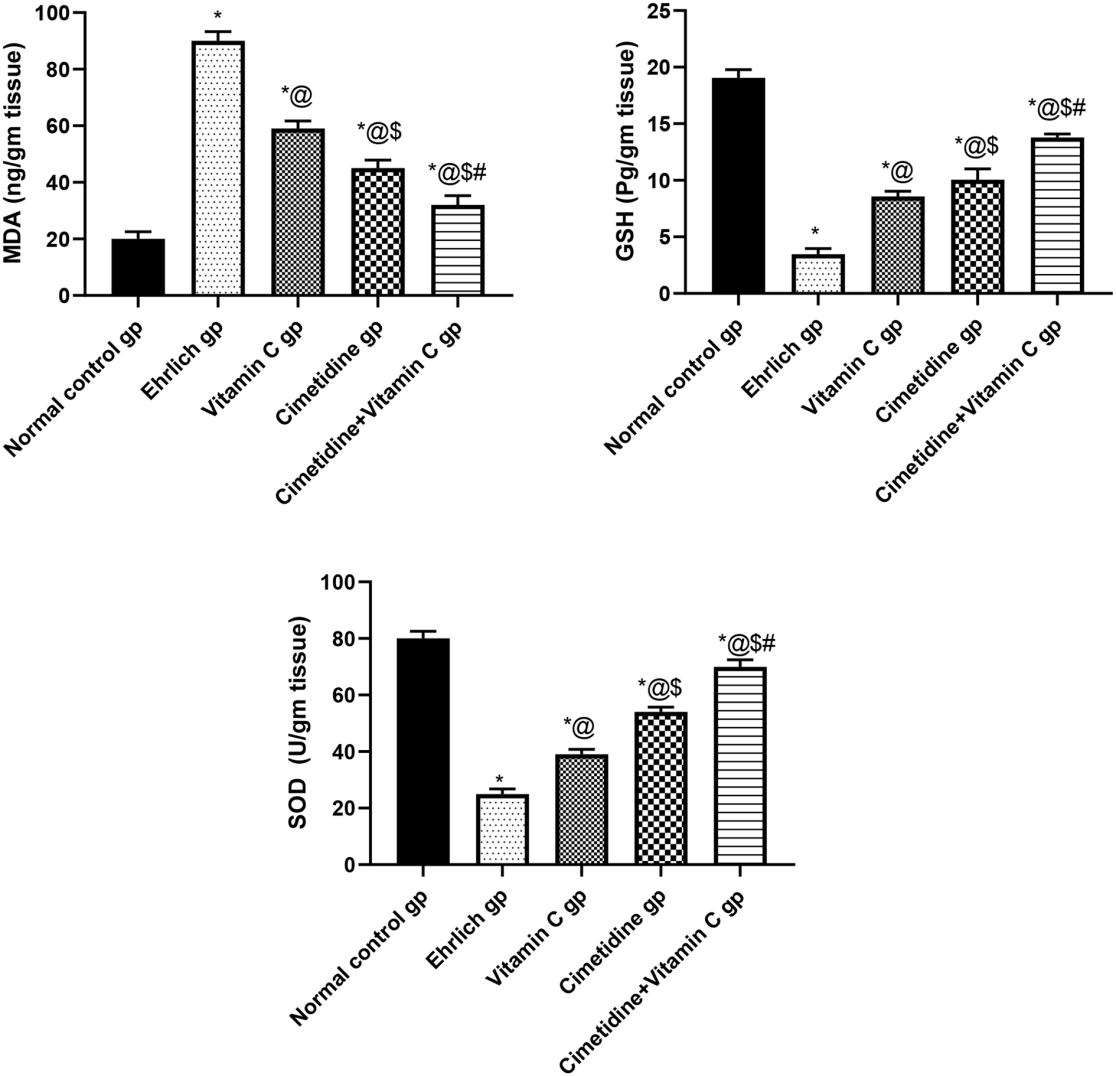
The changes in the tissue levels of oxidative stress parameters (MDA, GSH, SOD) following treatment with cimetidine at a dose of 200 mg/kg/day, vitamin C at a dose of 4 g/kg/day, and their combination for 15 days after Ehrlich induction of breast cancer in mice. Data are presented as mean ± SD.* is significantly different from the normal control group, P < 0.05. @ is significantly different from Ehrlich untreated group, P < 0.05. $ is significantly different from the vitamin C-treated group, P < 0.05. # is significantly different from cimetidine-treated group, P < 0.05.

The variation in the tumor tissue levels of oxidative stress parameters (MDA, SOD, GSH) after administration of cimetidine, vitamin-c, and a combination of both drugs is shown in (Fig. 6, and Supplementary 6). The results of our study showed that the MDA tumor level was significantly elevated by 4.5 folds compared to normal. The cimetidine caused a 50% decrease, while the vitamin C caused a 34.44% decrease in the MDA tumor level compared to untreated mice. Combining both of the previously mentioned drugs caused a 64.44% decrease in the MDA tumor level compared to untreated tumor-bearing mice.

Furthermore, GSH, and SOD tumor levels were significantly decreased by 81.53% and 68.75%, respectively, compared to their levels in the mammary fat pad of normal mice. The treatment with cimetidine, vitamin-c, and their combination caused a significant elevation in the GSH tumor level by 2.86, 2.45, and 3.94 folds, respectively. Moreover, the SOD tumor level was increased by 2.16 fold after cimetidine treatment, 1.56 fold after vitamin C treatment, and 2.8 folds after combination therapy compared to the untreated group.

##### Tissue level of TNF-α

**Figure 7:**
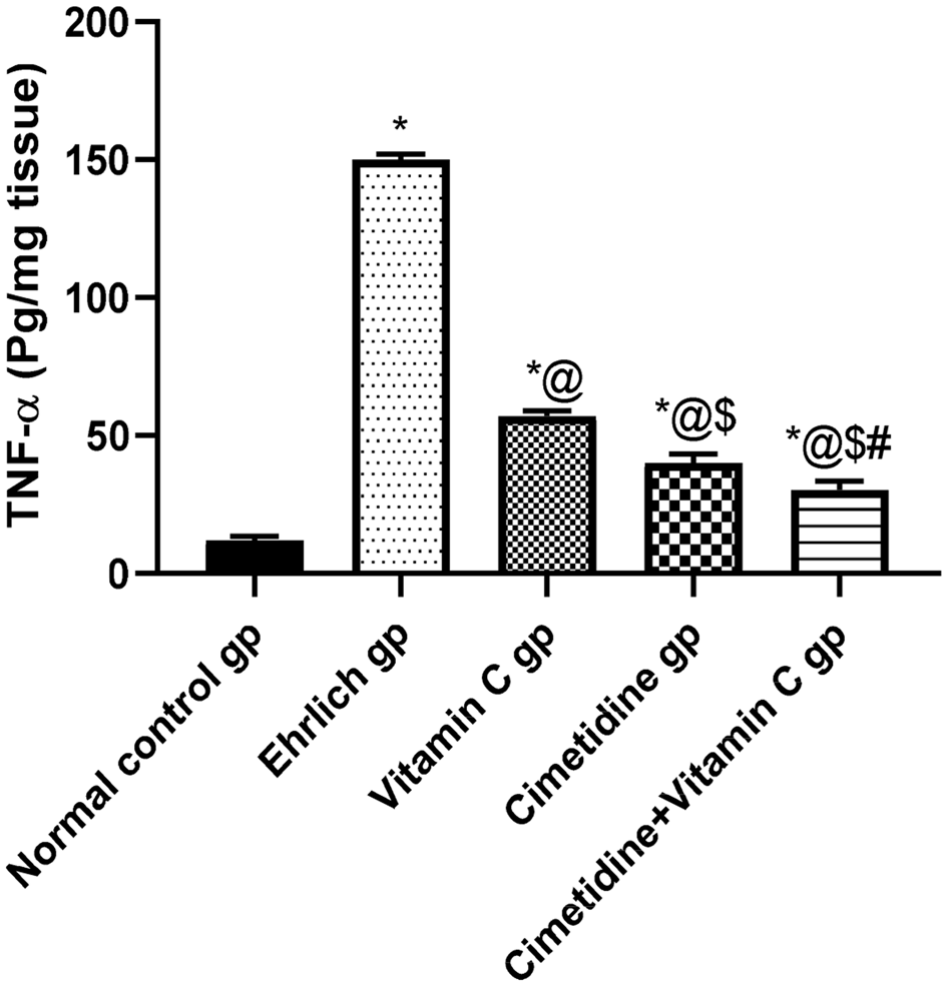
The changes in the tissue level of TNF-α following treatment with cimetidine at a dose of 200 mg/kg/day, vitamin C at a dose of 4 g/kg/day, and their combination for 15 days after Ehrlich induction of breast cancer in mice. Data are presented as mean ± SD.* is significantly different from the normal control group, P < 0.05. @ is significantly different from Ehrlich untreated group, P < 0.05. $ is significantly different from the vitamin C-treated group, P < 0.05. # is significantly different from the cimetidine-treated group, P < 0.05.

The change in the tumor tissue level of TNF-α in different groups was assessed to gain an insight into the inflammatory milieu (Fig. 7, and Supplementary 6). The results showed a remarkable increase in the tumor tissue level of pro-inflammatory TNF-α in the Ehrlich group, representing 1150% change from the normal control group. However, the cimetidine, vitamin C, and the combination of both drugs caused 73.33, 62, and 80% changes in the Ehrlich induced solid breast tumor. This illustrates that the combination therapy had the most significant effect on reducing the TNF-α tumor tissue.

##### Tissue level of histamine:

**Figure 8:**
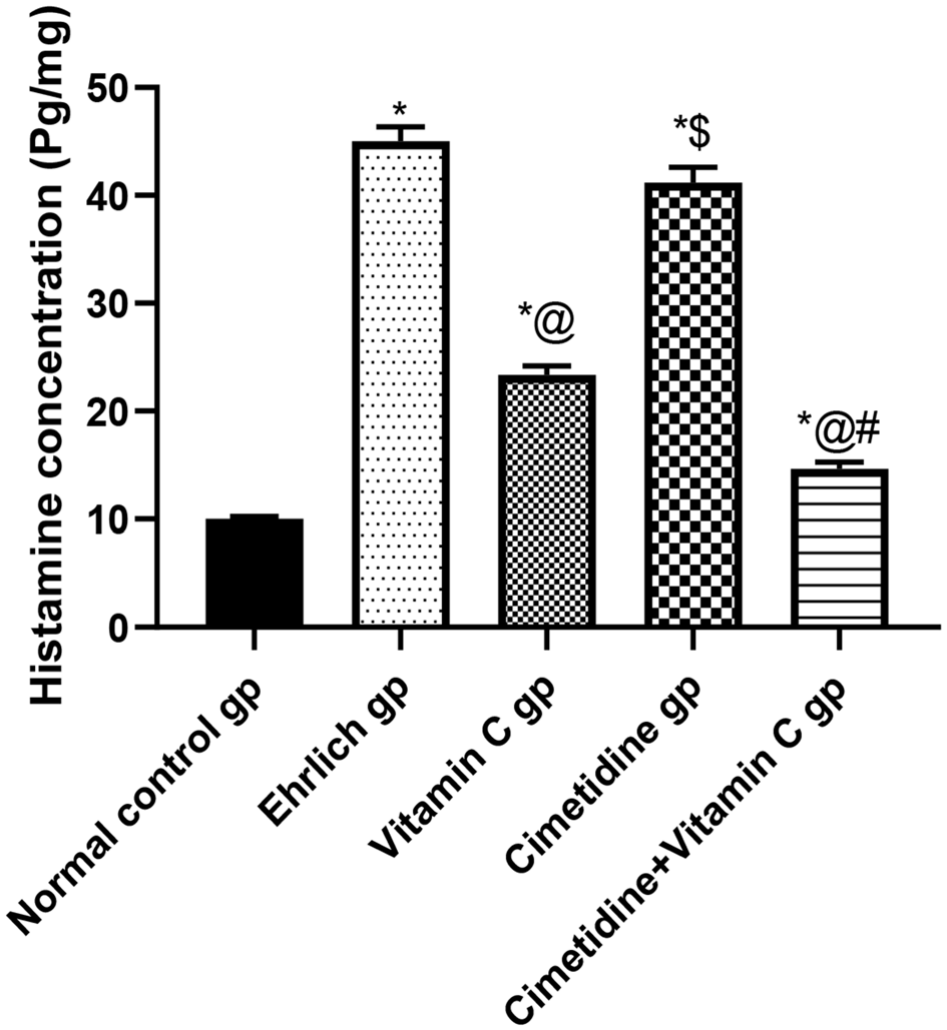
The changes in the tissue level of histamine following treatment with cimetidine at a dose of 200 mg/kg/day, vitamin C at a dose of 4 g/kg/day, and their combination for 15 days after Ehrlich induction of breast cancer in mice. Data are presented as mean ± SD.* is significantly different from the normal control group, P < 0.05. @ is significantly different from Ehrlich untreated group, P < 0.05. $ Significantly different from vitamin C- treated group, P < 0.05. # is significantly different from cimetidine-treated group, P < 0.05.

Figure 8, and Supplementary 6 illustrate the mammary and tumor microenvironment tissue levels of histamine in different groups, either untreated or treated with vitamin C and/or cimetidine. There was a significant increment in the histamine tissue level in untreated breast tumor-bearing mice, about 4.5 fold compared to the normal group. All given treatments could reduce the histamine tissue level except for cimetidine, and there was no significant difference from the untreated group. Moreover, there was no significant difference between the vitamin C and the combination-treated group.

##### Tissue level of cAMP

**Figure 9:**
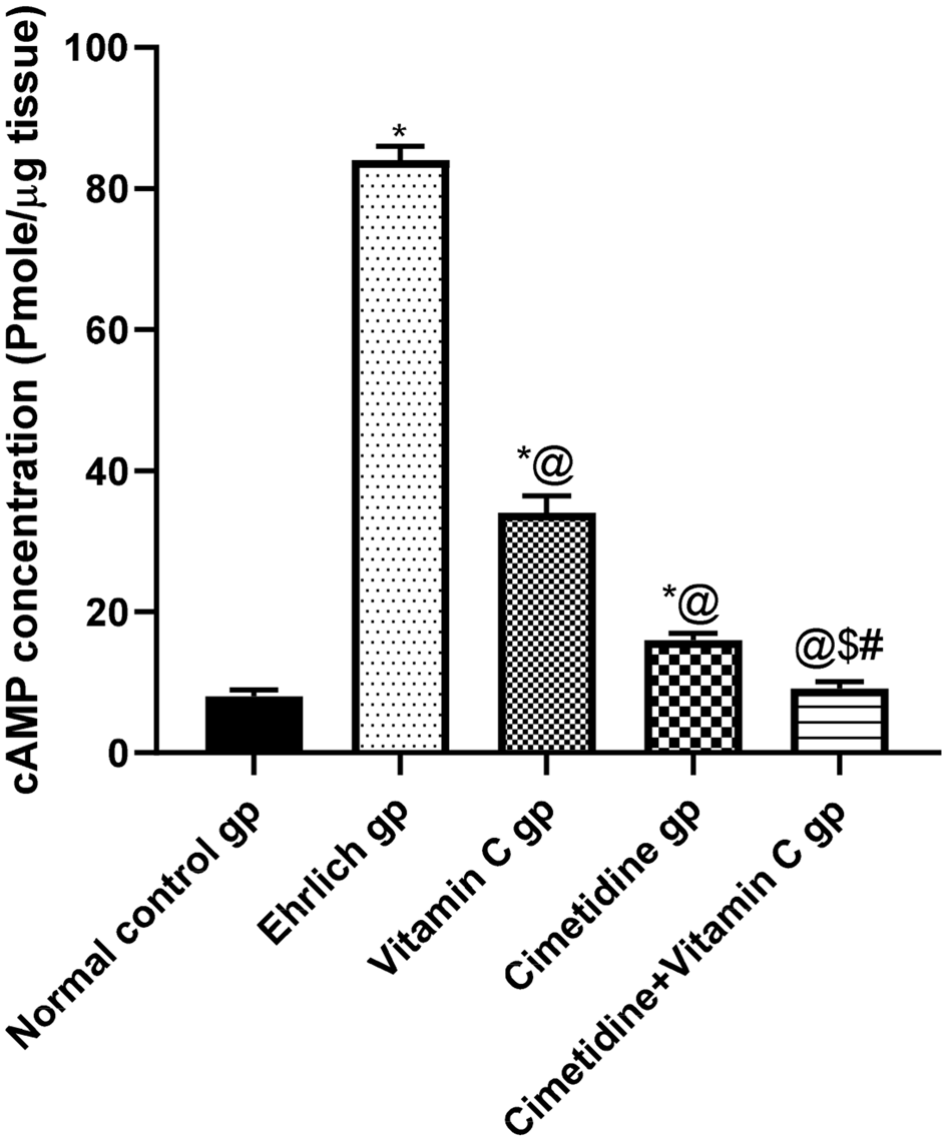
The changes in the tissue level of cAMP following treatment with cimetidine at a dose of 200 mg/kg/day, vitamin C at a dose of 4 g/kg/day, and their combination for 15 days after Ehrlich induction of breast cancer in mice. Data are presented as mean ± SD.* is significantly different from the normal control group, P < 0.05. @ is significantly different from Ehrlich untreated group, P < 0.05. $ is significantly different from the vitamin C- treated group, P < 0.05. # is significantly different from cimetidine-treated group, P < 0.05.

The breast and tumor tissue cAMP level in normal, Ehrlich induced breast mice, and different treated groups are shown in (Fig. 9, and Supplementary 6). It was observed that the tissue cAMP level was increased 10.5 folds in the Ehrlich induced mice compared to normal mice. All given treatments significantly mitigated the cAMP tissue level, with the most significant reduction seen in the combination therapy treated group,59.52% decrease for the vitamin C treated group, 64.33% decrease for the cimetidine treated group, and 89.17% decrease for the combination therapy treated group.

##### mRNA expression level of IRS-1

**Figure 10:**
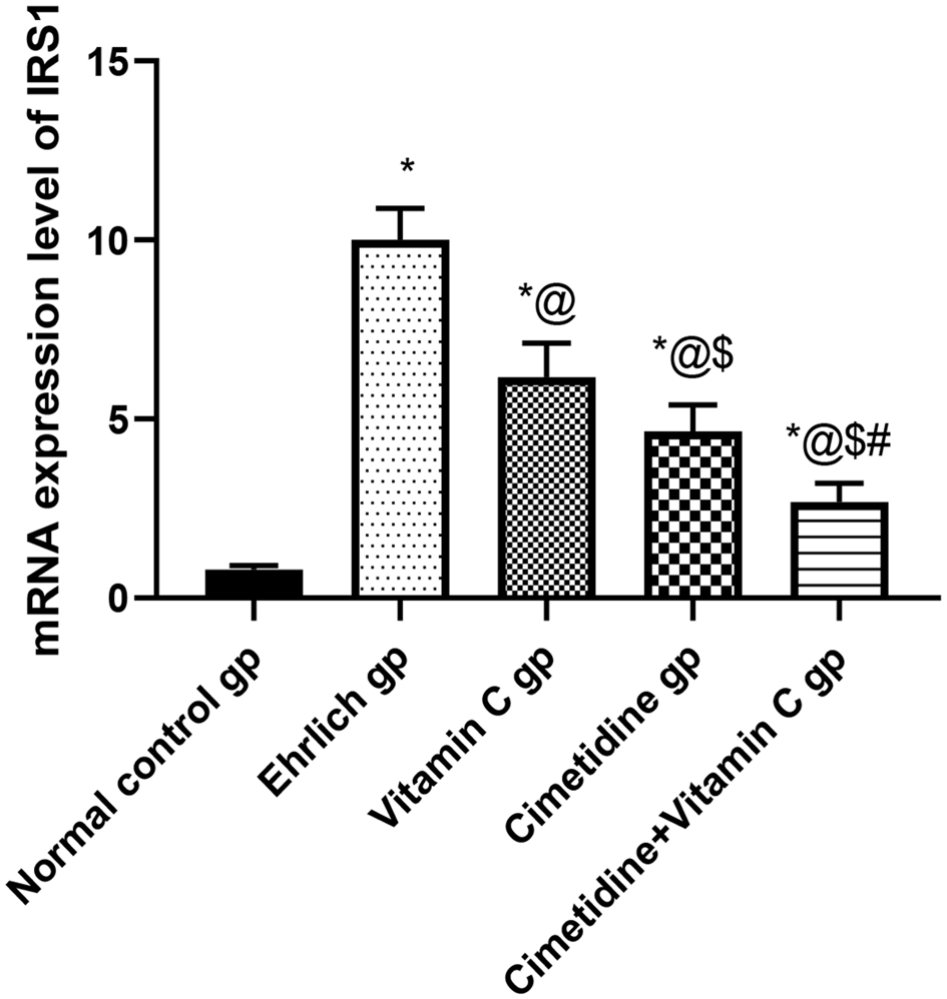
The relative mRNA expression level of IRS-1 following treatment with cimetidine at a dose of 200 mg/kg/day, vitamin C at a dose of 4 g/kg/day, and their combination for 15 days after Ehrlich induction of breast cancer in mice. Data are presented as mean ± SD.* is significantly different from the normal control group, P < 0.05. @ is significantly different from Ehrlich untreated group, P < 0.05. $ is significantly different from the vitamin C- treated group, P < 0.05. # is significantly different from cimetidine-treated group, P < 0.05.

To elucidate the mechanisms by which histamine produced by TAMC could modulate the PI3K/AKT/mTOR pathway, the mRNA expression of IRS-1 was evaluated (Fig. 10, and Supplementary 6). The relative mRNA of IRS-1 in the Ehrlich group was significantly elevated by 12.5 folds, respectively, compared to that of the normal control mice. The treatment with vitamin C significantly reduced the relative mRNA expression of the target gene by 38.3% compared to tumor-bearing untreated mice. Also, the cimetidine was capable of causing significant mitigation in the relative mRNA expression of IRS-1 by 53.5% compared to the mice in the Ehrlich group. Finally, the combination of both drugs caused the most significant decrease of the previously mentioned gene by 73.1%.

##### The PKA-C α/β, total and phosphorylated PI3K, AKT, mTOR expression levels in different groups by western blot technique:

**Figure 11:**
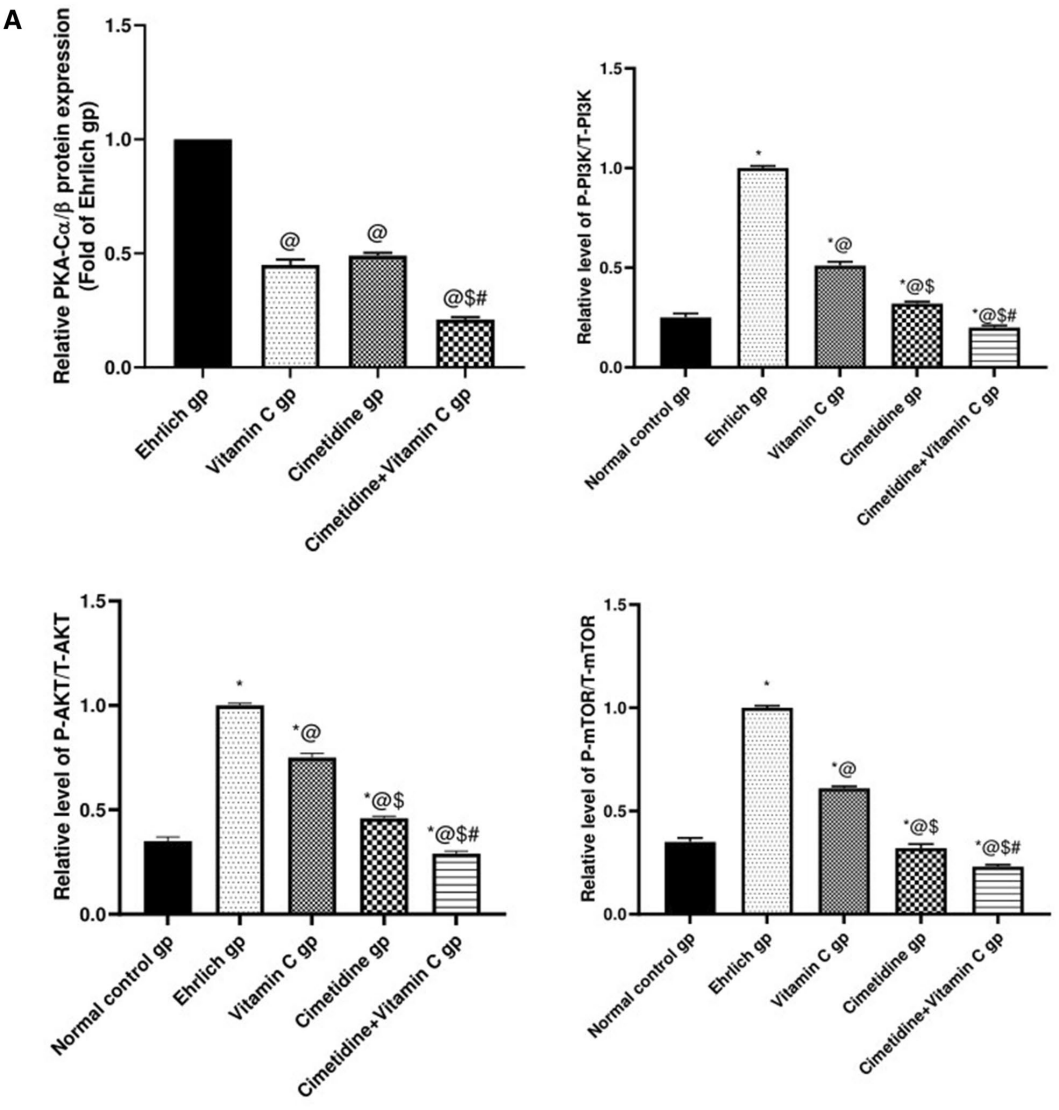

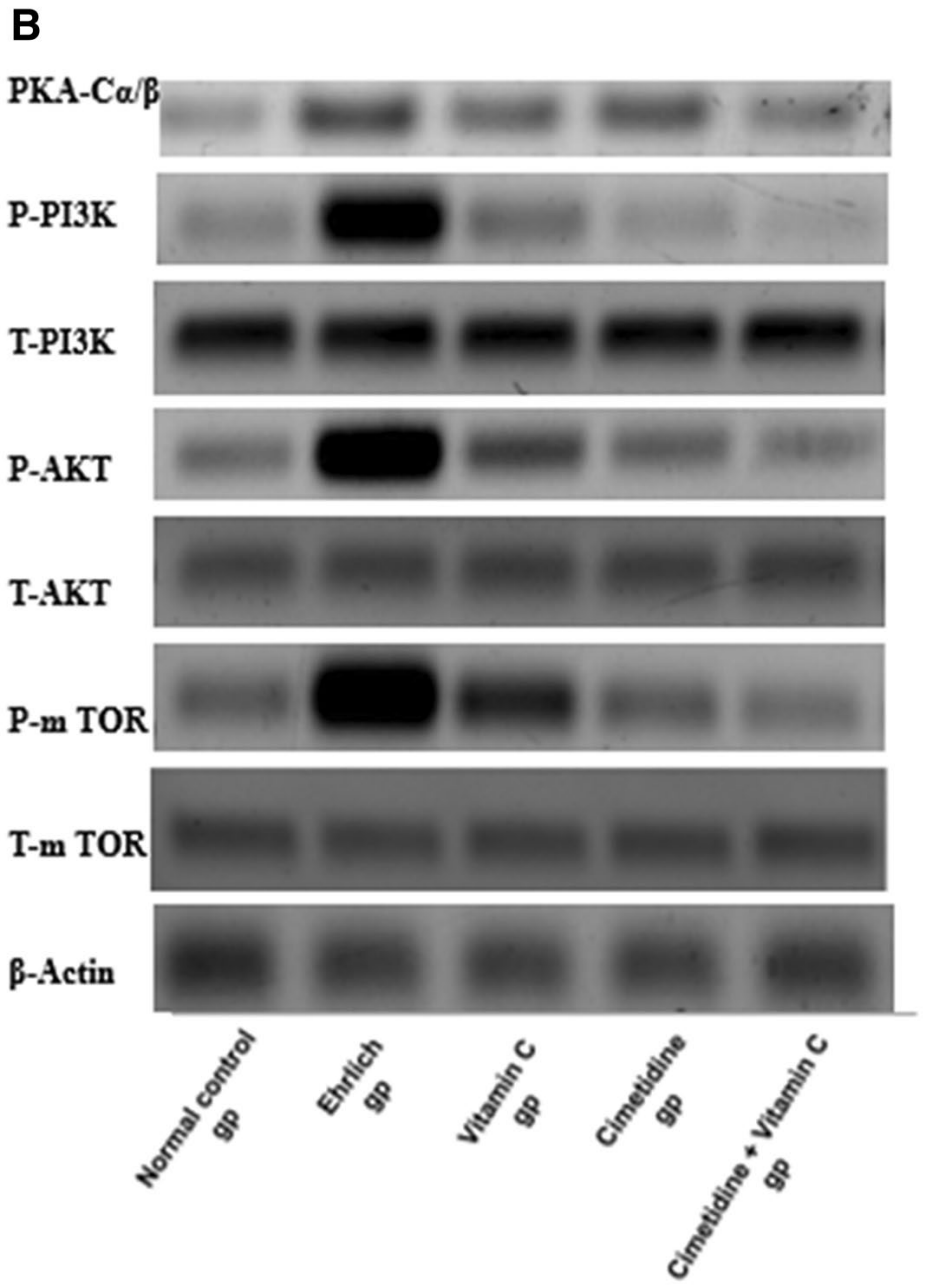
The changes in the PKA-C α/β, total and phosphorylated PI3K, AKT, mTOR expression levels after treatment with cimetidine at a dose of 200 mg/kg/day, vitamin C at a dose of 4 g/kg/day, and their combination for 15 days after Ehrlich induction of breast cancer in mice. Data are presented as mean ± SD.* is significantly different from the normal control group, P < 0.05. @ is significantly different from Ehrlich untreated group, P < 0.05. $ is significantly different from the vitamin C-treated group, P < 0.05. # is significantly different from cimetidine-treated group, P < 0.05.

(Fig. 11A,B, and Supplementary 1,2,3,4,5,7,8,9) illustrate the amendment in the protein expression level of PKA-C α/β, total and phosphorylated PI3K, AKT, and mTOR in different groups, which was carried out by the western blot technique. Firstly, the Ehrlich induced solid tumor in the mammary gland in untreated mice showed a significant elevation in the relative protein expression levels of PKA-C α/β, P-PI3K/T-PI3K, P-AKT/T-AKT, P-mTOR/T-mTOR, which play an important role in the pathogenesis of the disease. Furthermore, all given treatments in this study notably mitigated the relative expression of all of the aforementioned proteins. The effect of cimetidine was more obvious than that of vitamin C for all of the previously mentioned parameters except for PKA-C α/β. There was no significant difference between vitamin C and cimetidine-treated groups. Special emphasis on the effect of the combination therapy, which caused the most significant reduction in the previously mentioned proteins expression, modulating this pathway and mitigating their role in the pathogenesis of the disease.

##### Histopathological and histomorphometric assessment:

**Figure 12:**
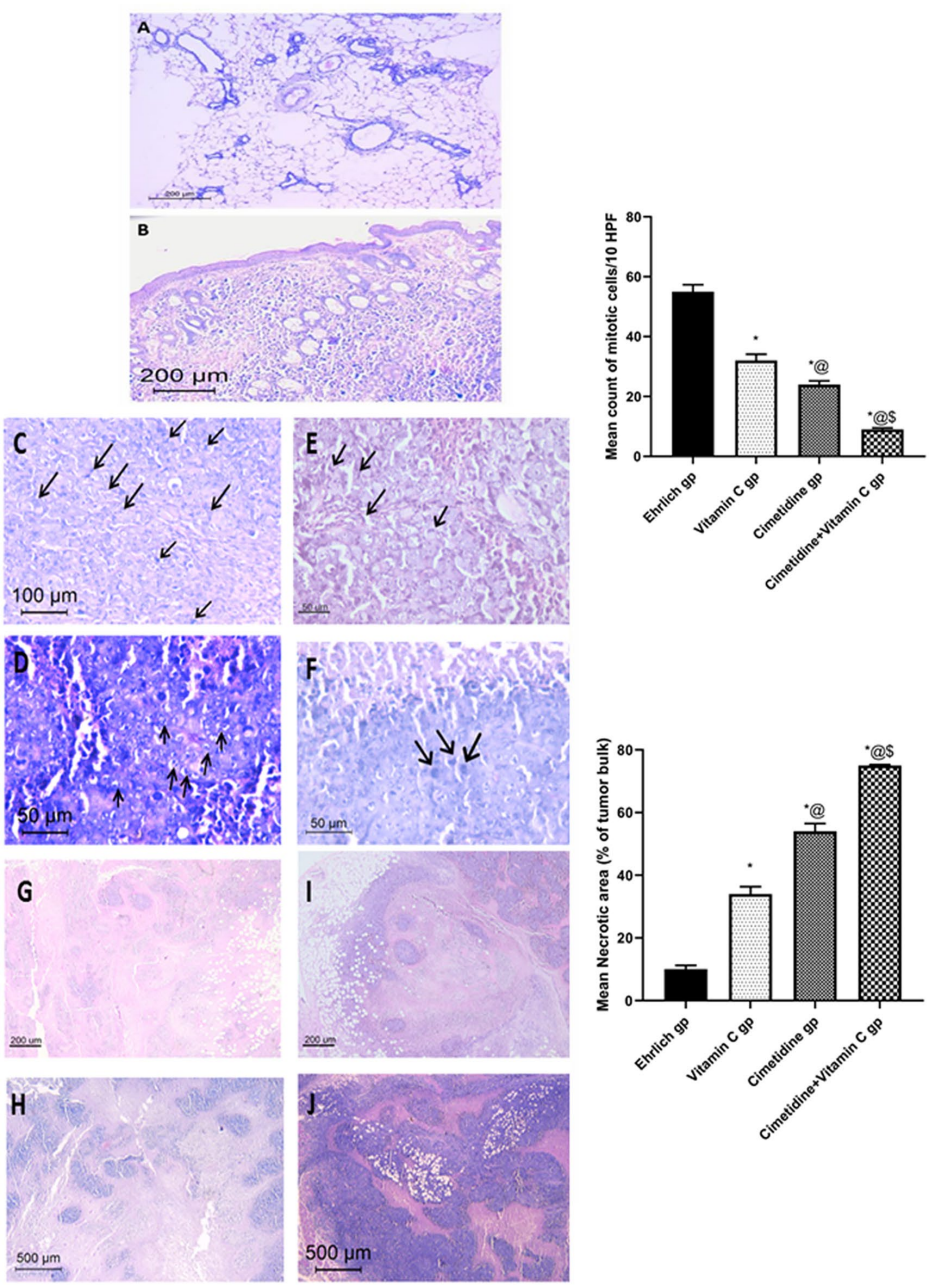
Histopathological and histomorphometric assessment using H and E for normal mammary fat pad and Ehrlich induced breast solid tumor in untreated mice and different treated groups with cimetidine at a dose of 200 mg/kg/day, vitamin C at a dose of 4 g/kg/day, and their combination for 15 days. Data are presented as mean ± SD.* Significantly different from the normal control group, P < 0.05. @ is significantly different from Ehrlich untreated group, P < 0.05. $ is significantly different from the vitamin C-treated group, P < 0.05. # is significantly different from the cimetidine-treated group, P < 0.05. (**A**) A normal control mice breast shows normal breast lobules and ducts. (**B**) Ehrlich tumor invades the entire breast tissue. (**C**) Untreated Ehrlich tumor shows numerous mitotic figures. (**D**) Mild reduction in mitotic figures is shown in breast solid tumor after treatment with vitamin C. (**E**) Moderate reduction in mitotic figures is shown in breast solid tumor after treatment with cimetidine. (**F**) The most prominent reduction is shown in breast solid tumor after treatment with the combination of cimetidine and vitamin C. (**G**) Ehrlich tumor is composed of sheets and masses of large polygonal epithelioid cells, with central areas of necrosis. (**H**) Demonstrates mild elevation in the extent of necrosis within the tumors after vitamin C treatment. (**I**) Reveals moderate elevation in the extent of necrosis within the tumors after cimetidine treatment. (**J**) Indicates the most prominent elevation in the extent of necrosis within the tumors after treatment with the combination therapy.

Haematoxylin and eosin (H and E) stain was used to assess the histopathological changes in the normal breast mammary tissues compared to breast solid tumor induced by Ehrlich and the effect of different drug treatments (Fig. 12). The normal mammary fat pad examination revealed normal breast lobules and ducts (Fig. 12A). On the other hand, an assessment of the induced group showed aggressive infiltrative tumors composed of sheets, masses, and cords of polygonal, cohesive cells with enlarged irregular vesicular nuclei having prominent nucleoli and ample eosinophilic to amphophilic cytoplasm (Fig. 12B).

In the Ehrlich induced breast cancer tissues of untreated mice, the mitotic figures were numerous, and reaching mean counts of (55/10 HPFs) (Fig. 12C). Treatment with vitamin C significantly reduced the mitotic rate reaching mean counts of (32 /10 HPFs), P-value < 0.05, Fig. 12D. Also, cimetidine-treated mice showed more significant reduction in the mitotic rate, reaching mean counts of (24/10 HPFs), P-value < 0.05, Fig. 12E. Moreover, the combination treatment caused the most observed significant reduction in the mitotic figures reaching mean counts of (9/10 HPFs), P-value < 0.05 (Fig. 12F).

Small areas of necrosis were noted within the center of the tumors in the solid breast cancer-bearing mice (mean area 10% of the tumor bulk) (Fig. 12G).

Treatment with vitamin C caused mild elevation in the necrotic areas (mean area 34% of the tumor bulk) (Fig. 12H). At the same time, the cimetidine showed a moderate increment in the necrotic areas (mean area 54% of tumor bulk) (Fig. 12I). In the combination therapy-treated mice, tumors showed the largest areas of necrosis (Mean area 75% of the tumor bulk), P-value < 0.05 (Fig. 12J). All increased areas of necrosis were mainly noted to be on the borders of the tumor tissues.

##### Immunohistochemistry

**Figure 13:**
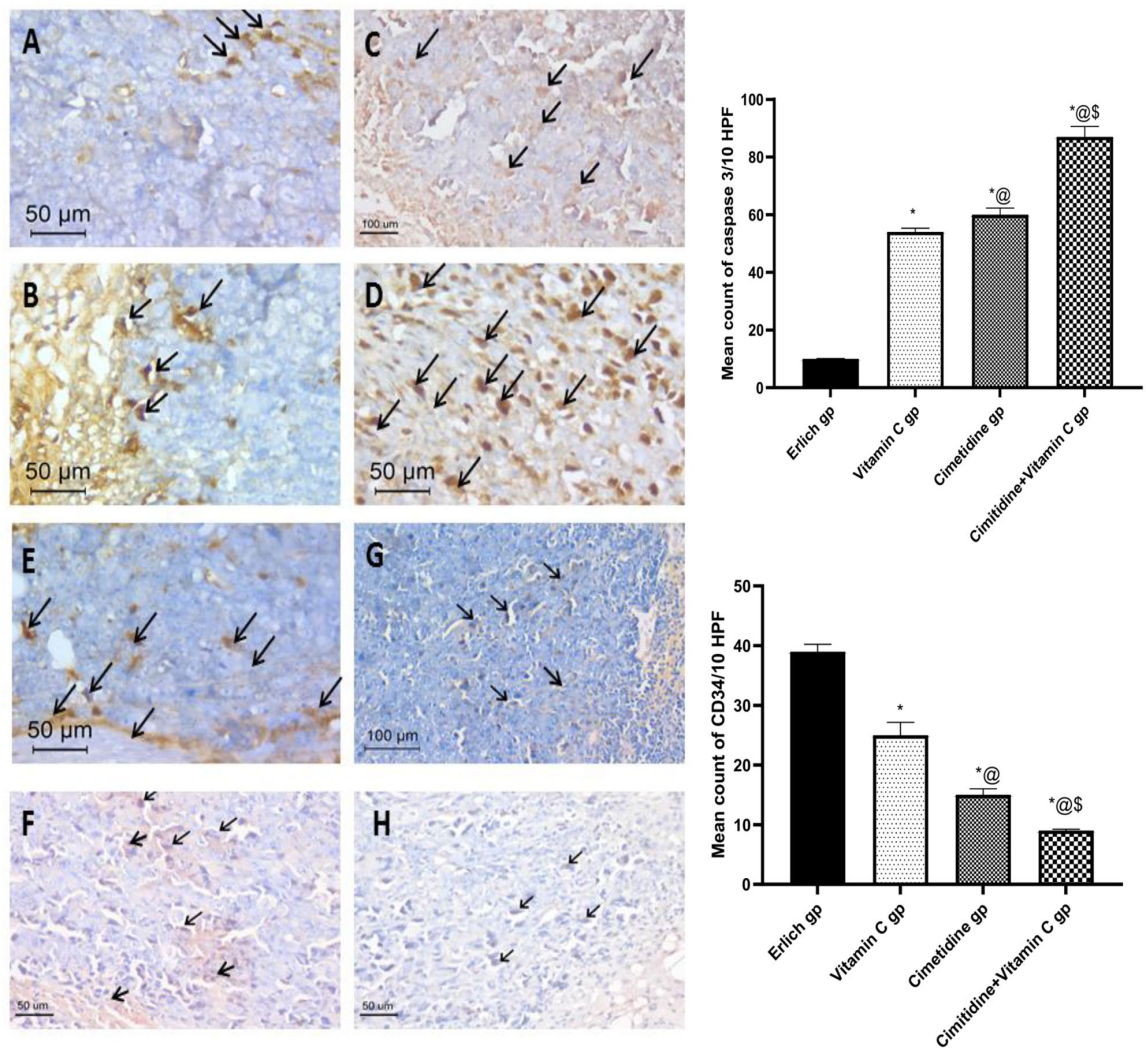
The changes in the caspase 3 and CD34 immunostain in Ehrlich induced breast solid tumor in untreated mice and different treated groups with cimetidine at a dose of 200 mg/kg/day, vitamin C at a dose of 4 g/kg/day, and their combination for 15 days. Data are presented as mean ± SD.* Significantly different from the normal control group, P < 0.05. @ is significantly different from Ehrlich untreated group, P < 0.05. $ is significantly different from the vitamin C- treated group, P < 0.05. # is significantly different from the cimetidine-treated group, P < 0.05. (**A**) shows caspase 3 immunostain within the tumor of untreated mice. (**B**–**D**) Shows caspase 3 immunostain within the tumor after treatment with vitamin C, cimetidine, and the combination therapy, respectively. (**E**) shows CD34 immunostain within the tumor of untreated mice. (**F**–**H**) reveals CD34 immunostain within the tumors after treatment with vitamin C, cimetidine, and the combination therapy, respectively.

Immunohistochemical staining for Caspase 3 and CD34 is shown in (Fig. 13). This immunostaining revealed the extent of apoptosis and angiogenesis in the solid breast tumor after treatment with vitamin C and/or cimetidine. The mean count of caspase 3 in the untreated breast cancer in mice was 10/10HPF (Fig. 13A). The vitamin C treatment mildly increased the level of caspase 3 in the breast tumor, reaching a mean count of 54/10 HPF (Fig. 13B). The cimetidine treatment moderately increased the level of caspase 3 in the breast tumor, reaching a mean count of 60/10 HPF (Fig. 13C). The combination therapy caused the most prominent increase in the level of caspase 3 in the breast tumor, reaching a mean count of 87/10 HPF (Fig. 13D).

Staining for CD34 highlighted the extensively formed tumor vessels, particularly at the invasive tumor front (mean count of 39/10HPF) (Fig. 13E). The vitamin C treatment caused a mild decrease in the CD34, reaching a mean count of (25/10HPF) (Fig. 13F). The cimetidine treatment caused a moderate reduction in angiogenesis than vitamin C, reaching a mean count of 15/10HPF (Fig. 13G). The combination therapy caused the most significant reduction in the CD34, reaching a mean count of 9/10HPF (Fig. 13H).

### Statistical correlations

**Figure 14:**
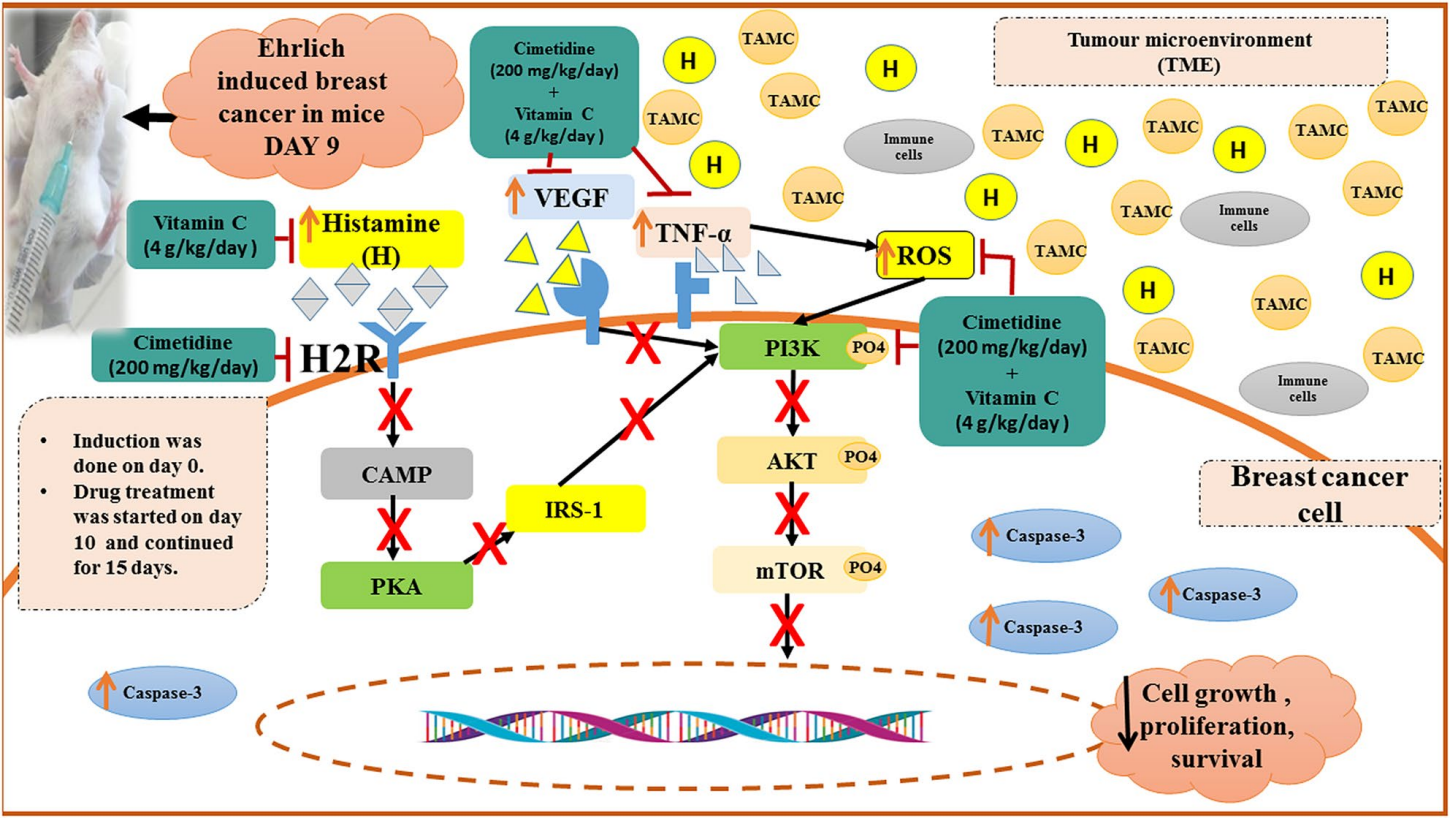
Shows the role of tumor-associated mast cells and their mediators (histamine, VEGF, and TNF-α) within the TME as well as the proposed mechanism of the anti-tumor effect of Cimetidine/Vitamin C by modulating mast cells mediators resulting in the restoring of oxidative stress balance, inhibition of angiogenesis and inactivation of PI3K/AKT/mTOR pathway leading to cancer cells apoptosis. *TAMC* tumour associated mast cell, *TME* tumour microenviroment, *ROS* reactive oxygen species, *H2R* histamine receptor 2, *VEGF* vascular endothelial growth factor, *TNF-α* tumour necrosis factor alpha, *cAMP* cyclic adenosine monophosphate, *PKA* protein kinase A, *IRS-1* insulin receptor substrate-1, *PI3K* phosphatidylinositol-3-kinase, *AKT* serine/threonine kinase-1, *m-TOR* mammalian target of rapamycin.

Statistical correlations were performed using the Spearman coefficient test to investigate the association between mast cell mediators (histamine, VEGF, and TNF-α) and members of the PI3K/AKT/mTOR pathway, apoptosis, and angiogenesis within cimetidine and vitamin C treated group. The results of the statistical correlations revealed a positive correlation between mast cell mediators (histamine, VEGF, and TNF-α) and each of PI3K, AKT, mTOR, PKA, IRS1, and CD 34. On the other hand, a negative correlation was observed between mast cell mediators (histamine, VEGF, and TNF-α) and caspase-3 levels within the TME (Table 5). The proposed mechanisms of the correlations and the proposed mechanism of action of cimetidine and vitamin C are summarized in (Fig. 14).

**Table 5:** Statistical correlations between mast cell mediators (histamine, VEGF, and TNF-α) and members of the PI3K/AKT/mTOR pathway, PKA, IRS-1, CD34 and caspase 3 in the cimetidine/vitamin c treated group.

	Histamine	VEGF	TNF
PI3K	rs = 0.93, p < 0.01	rs = 0.83, p = 0.009	rs = 0.87, p = 0.004
AKT	rs = 0.91, p = 0.001	rs = 0.77, p = 0.02	rs = 0.89, p = 0.002
mTOR	rs = 0.8, p = 0.01	rs = 0.92, p < 0.01	rs = 0.67, p = 0.06
PKA	rs = 0.72 , p = 0.04	rs = 0.71, p = 0.04	rs = 0.74, p = 0.03
IRS1	rs = 0.8, p = 0.01	rs = 0.71, p = 0.04	rs = 0.76, p = 0.02
Caspase 3	rs = − 0.8, p = 0.01	rs = − 0.89, p = 0.002	rs = − 0.61, p = 0.1
CD 34	rs = 0.89, p = 0.002	rs = 0.9, p = 0.001	rs = 0.81, p = 0.01

## Discussion

Breast cancer is the most frequent malignancy in women and a common cause of death worldwide. While early stages of breast cancer are associated with a good prognosis, the survival rate rapidly declines once the tumor metastasizes and spreads to distant organs. The conventional treatment options for breast cancer are effective but comprise many side effects that patients cannot bear in many cases. Recent approaches in research aim to find potential pharmacological substitutions to conventional treatments of breast cancer to improve the quality of life of these patients^1^.

The TME is a promising target for cancer therapy because its resident cells play a critical role in defining the tumor immune response, either by promoting tumor growth and proliferation or by enhancing the anti-tumor effect of the immune effector cells. Mast cells and their mediators have previously been linked with tumor progression and metastasis, where histamine, the primary mast cell mediator, and its receptors (HR1-HR4) were upregulated in many cancers and associated with cancer survival metastasis and recruitment of suppressive cells to the TME^4^.

Cimetidine (CIM) is an H_2_RA used to accelerate the healing of gastrointestinal ulcers. In previous studies, it has been demonstrated to possess anti-tumor activity against colon, gastric and kidney cancers, and melanomas^13^. Ascorbic acid is a water-soluble vitamin and a potent antioxidant drug utilized to treat many conditions^23^. Many previous studies illustrated its effectiveness in delaying tumor growth in humans and murine tumor models and its ability to act as a mast cell stabilizer^24–26^.

The drugs used in this study were chosen based on these previous findings. Our primary objective was to determine the anti-tumor activity of cimetidine, vitamin C and their combination via targeting TAMC and its mediators (histamine, VEGF, and TNF-α) in the TME and investigating their effect on PI3K/AKT/mTOR cue in Ehrlich induced breast cancer in mice.

Ehrlich induced solid breast cancer model in mice was chosen due to its simplicity, short duration of the induction method, and closeness to human breast cancer pathogenesis^27^.

Regarding TAMC mediators' levels within the TME, the current data revealed an increment in the histamine level in the tumor of untreated mice compared to the normal mammary fat pad, which was consistent with previous studies^28,29^. The efficacy of cimetidine in decreasing the histamine level within the TME was significantly lower than that observed in the vitamin C and the combination therapy groups. The most substantial reduction was noticed in the combination therapy treated group. It has been previously reported that cimetidine (H2R antagonist) mitigated the effect of histamine in breast cancer and melanoma cell lines^30^; however, other histamine receptors play a vital role within the TME. The HR4 was overexpressed on TAMC and critical in histamine-mediated mast cells recruitment to the TME. Therefore, in this case, cimetidine has not the ability to prevent mast cells recruitment^31^. In contrast, vitamin C functions differently; it acts as a mast cell stabilizer and inhibits histidine decarboxylase, the enzyme essential for histamine production. Finally, it upregulates histamine degradation by increasing the level of diamine oxidase^15^.

Herein, we could suggest that the expected increase in the mast cell count in the breast tumors of untreated mice was not only responsible for the elevation in the histamine level but also an increase in VEGF and angiogenesis levels in untreated breast tumors in mice. Both elevated parameters were implicated in the pathogenesis and progression of breast cancer^32^.

Angiogenesis augments tumor growth and proliferation. Therefore, the serum levels of VEGF and count of CD34 were assessed as the primary markers of angiogenesis. The current results showed a considerable increase in the VEGF serum levels and count of CD34 in tumor tissues in untreated mice compared to normal, which corroborate previous studies^20,33,34^. All treatments in our study caused a major decrease in the elevated serum levels of VEGF and tissue count of CD34 in Ehrlich-induced breast cancer in mice. Furthermore, previous results illustrated that the increased accumulation of mast cells led to histamine release, contributing to angiogenesis in the carrageenan-induced VEGF protein overexpression and granulation tissue in rats via the H_2_R/cAMP/PKA pathway^35,36^.

Moreover, cimetidine was shown to inhibit angiogenesis in the granulation tissue in the aforementioned model^37^. Additionally, cimetidine was found to significantly decrease both serum and tissue levels of VEGF in Colon 38-bearing mice^38^. The capability of vitamin C to incline the VEGF production in B16F10 murine melanoma cells and the Xenograft Model of Colon Cancer was demonstrated^39,40^. Herein, the combination therapy of cimetidine and vitamin C caused the most significant decline in the serum levels of VEGF and expression of CD34 in breast cancer tissues, illustrating its promising anti-angiogenic effect in treating breast cancer.

TNF-α, the third TAMC mediator studied, is not only a pro-inflammatory cytokine produced by the inflammatory cells in the TME, but also a pro-tumorigenic agent. The present findings reveal that Ehrlich-induced breast cancer provoked the tumor TNF-α levels, while cimetidine, vitamin C, and their combination reduced the aforementioned pro-inflammatory cytokine level. The most significant reduction was observed in the combination-treated group, emphasizing this therapy's role in mitigating inflammation and preventing activation of further molecular mechanisms that are enrolled in breast cancer progression^41^. It has been reported that vitamin C tackled TNF-α in Severe Community-Acquired Pneumonia patients and LPS-induced Macrophages cells^42^. Cimetidine pretreatment to alcohol intake in rats reduced the gastric mucosal TNF-α level compared to alcohol-treated rats^43^. TNF-α role within the TME is versatile and supports tumor growth in many mechanisms. It was found to possess pro-angiogenic properties. The statistical correlations revealed a positive association between the three TAMC mediators and the CD34 angiogenesis marker. Further, TNF-α was observed to have the ability to augment the oxidative stress within the TME, which leads to increased proliferation and recruitment of inhibitory immune cells within the TME, such as regulatory T cell (Treg) and myeloid-derived suppressor cells (MDSC) and apoptosis of effector T cells, thus supporting tumor immune evasion^44^.

Further, oxidative stress status was shown to play a vital role in the pathogenesis of breast cancer disease, occurring as a result of shifting the balance to word prooxidants than antioxidants^8^. This study showed a tremendous increase in the untreated mice breast cancer levels of MDA, which was in agreement with the results of previous studies^45,46^. Furthermore, the results of the tumor level of SOD and GSH in the untreated breast cancer-bearing mice revealed a very significant decrease compared to the mammary fat pad of normal mice.

In agreement with earlier work, Cimetidine ameliorated the oxidative stress from low-dose cumulative irradiation by elevating the activity of SOD and GSH enzymes and tackling the MDA levels^47^. The antioxidant effect of vitamin C has also been reported in many investigations^48,49^.

Interestingly, the combination caused the most significant halting of the elevated MDA levels and elevated the SOD and GSH levels in breast cancer tissues, illustrating its beneficial role in reverting the state of oxidative stress.

The PI3K/AKT/mTOR pathway was assessed to reveal the signaling mechanisms by which the TAMC mediators support tumor growth and survival. It was suggested that the elevated levels of histamine could activate H2R leading to an increase in the level of cAMP, which is subsequently accompanied by activation of PKA^7^. This suggestion was in line with our results, showing an elevation in both histamine tumor levels and mRNA expression of PKA in untreated tumor-bearing mice. As an H2R blocker, Cimetidine tackled the cAMP-PKA activation, as confirmed by reduced expression levels of PKA in the cimetidine-treated mice. Vitamin C was also capable of reducing the activation of PKA, which was suggested to decrease the histamine level and subsequently reduce the activation of H2R. The most apparent reduction in PKA activity was observed in the combination-treated group.

The PKA can interact with downstream IRS-1 to activate the PI3K/AKT/mTOR pathway related to cancer cell survival, proliferation, and growth^11^. Our results indicated that the activated PKA in untreated tumor-bearing mice could activate IRS-1, as evidenced by increased mRNA expression levels of IRS-1. Furthermore, a significant increase in the expression of PI3K, AKT molecules was shown in the aforementioned group of mice, suggesting that PKA subsequently activated IRS-1, which was capable of stimulating PI3K/AKT cue in the untreated breast cancer-bearing mice. The combination therapy of cimetidine and vitamin C mainly inhibited PKA/IRS-1/PI3K/AKT activation. Furthermore, the PI3K/AKT could be positively regulated by VEGFRs and their ligands^50^.

Moreover, it has been observed that oxidative stress status in breast cancer could activate the PI3K/AKT/mTOR pathway via mitigating the activation of PTEN, and the pathway's negative regulator^51^. Thus, we may assume that the combination therapy could also inhibit the activation of the PI3K/AKT/mTOR pathway by reducing histamine levels and tackling the elevated levels of VEGF and ROS.

Further, statistical correlation conducted to support the findings and the proposed pathway of the current study revealed that TAMC mediators (histamine, VEGF, and TNF-α) are positively correlated with the expression of PI3K, AKT, mTOR, and IRS1, indicating their role in tumor proliferation and survival by the activation of PI3K/AKT/mTOR cue.

Tumor volume was measured to assess the inhibitory effect of the given treatments on breast tumor growth. In earlier studies, cimetidine has been reported to inhibit hepatocellular carcinoma growth and the growth of transplantable human gastric cancer SGC-7901 cells in vivo^52,53^. Furthermore, vitamin C previously reduced tumor volume in bladder cancer in vivo and mitigated the growth of ovarian, pancreatic, and glioblastoma tumors established in mice^26,54^. Our results showed that ability of cimetidine treatment to reduce the Ehrlich induced breast tumor volume in mice was more than that observed in the vitamin C treated group. The combination therapy caused the most significant decrease in the tumor volume. These results pointed to the beneficial effect of the combination in maximally inhibiting breast tumor growth.

CEA level, mitosis, necrosis, and Caspase 3 levels were assessed to confirm the anti-tumor effect of the drugs under study. CEA is a tumor marker that is widely used in monitoring breast cancer. In the untreated breast cancer-bearing mice, the serum level of CEA was considerably elevated compared to normal control mice, which is consistent with the results of an earlier study^2^. Cimetidine, vitamin C, and their combination reverted the elevated serum level of CEA, with the most pronounced reduction occurring in the combination-treated group. A previous in vivo study confirmed the beneficial effect of vitamin C in lowering the level of CEA in colon cancer. The same effect was also observed in colon cancer patients after being treated with high dose intravenous vitamin C^55,56^.

Mammary fat pads from normal mice were examined histologically and histomorphometrically and indicated normal breast lobules and ducts. While the examination in the Ehrlich group showed aggressive infiltrative tumors cells, numerous mitotic figures and areas of necrosis were minor. All given treatments significantly increased the areas of necrosis around the tumor and decreased the count of mitotic figures. The most significant effect was observed in the combination therapy treated group, emphasizing the beneficial role of the combination therapy in tackling the mitotic process and promoting tumor cell necrosis.

Caspase 3 is the critical regulator of apoptotic cell death. The immunohistochemistry results indicated that the level of caspase 3 in the untreated mice was significantly reduced compared to the normal mice, which promotes tumor cell survival, as demonstrated in a previous study^57^. All the treatments given caused a remarkable increment in the level of caspase. The highest elevation was observed in the combination-treated group, revealing the beneficial role of the combination in promoting apoptosis and reducing the survival of the tumor cells. Cimetidine was previously shown to trigger apoptosis in gastric cancer cells in vitro, which corroborates our findings^53^. Vitamin C was also reported to increase the extent of apoptosis via activation of caspase 3 in Oral Squamous Cell Carcinoma^58^. The efficacy of the current treatment options to induce apoptosis is proposed to be due to their effectiveness in inhibiting the production of the TAMC mediators and thus reversing their survival effects. The statistical correlations revealed a negative correlation between TAMC mediators (histamine, VEGF, and TNF-α) and Caspase 3 level within the TME. Moreover, vitamin C apoptosis induction has been demonstrated via different mechanisms, including activation of NK cells, increased FAS-induced apoptosis via effector T cells, or increased mitochondrial permeability, leading to cancer cell death^59^.

The above findings in the current study suggested the beneficial role of the Cimetidine/Vitamin C combination as an anti-tumor immunomodulatory therapy for breast cancer. The in-vitro study proves the synergistic effects of the co-administration of the drugs under investigation.

## Conclusion

Optimistically, we could conclude that the cimetidine and vitamin C therapy mitigated Ehrlich-induced breast solid tumor in mice via reducing angiogenesis, oxidative stress, inflammation. Furthermore, they were capable of tackling the TAMC mediators and inhibiting the activation of PI3K/AKT/mTOR cue. Moreover, the synergistic effect of the cimetidine/vitamin C combination inhibited the proliferation of tumor cells and promoted tumor cell death via apoptosis and necrosis, which decreased tumor survival. This suggests its use as one of the treatment options for breast cancer (Supplementary Information).

## Supplementary Information

Supplementary Information 1.

Supplementary Information 2.

Supplementary Information 3.

Supplementary Information 4.

Supplementary Information 5.

Supplementary Information 6.

Supplementary Information 7.

Supplementary Information 8.

Supplementary Information 9.

## Data Availability

All data generated or analyzed during this study are included in this published article.
