# Supplementary figures and images for: Anti-neoplastic action of Cimetidine/Vitamin C on histamine and the PI3K/AKT/mTOR pathway in Ehrlich breast cancer
Source: Sci Rep. 2022 Jul 7;12:11514. doi: 10.1038/s41598-022-15551-6 (PMC9262990; doi:10.1038/s41598-022-15551-6)

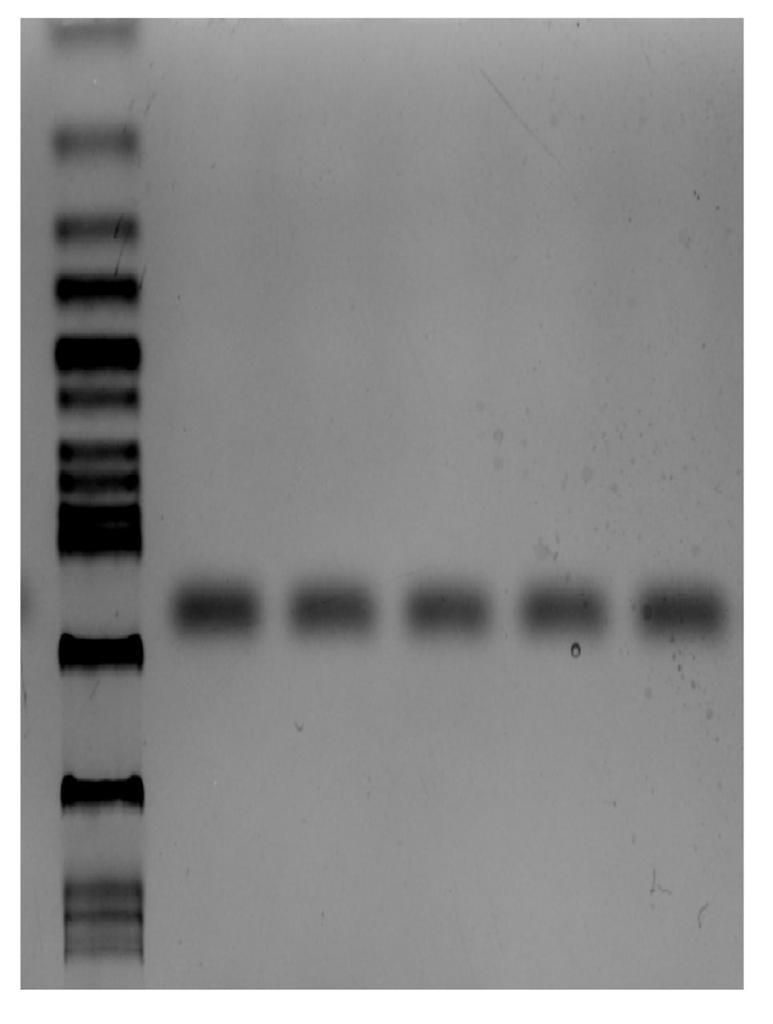

Supplement: Supplementary file 1 — Supplementary Information 1. [file 41598_2022_15551_MOESM1_ESM.jpg]

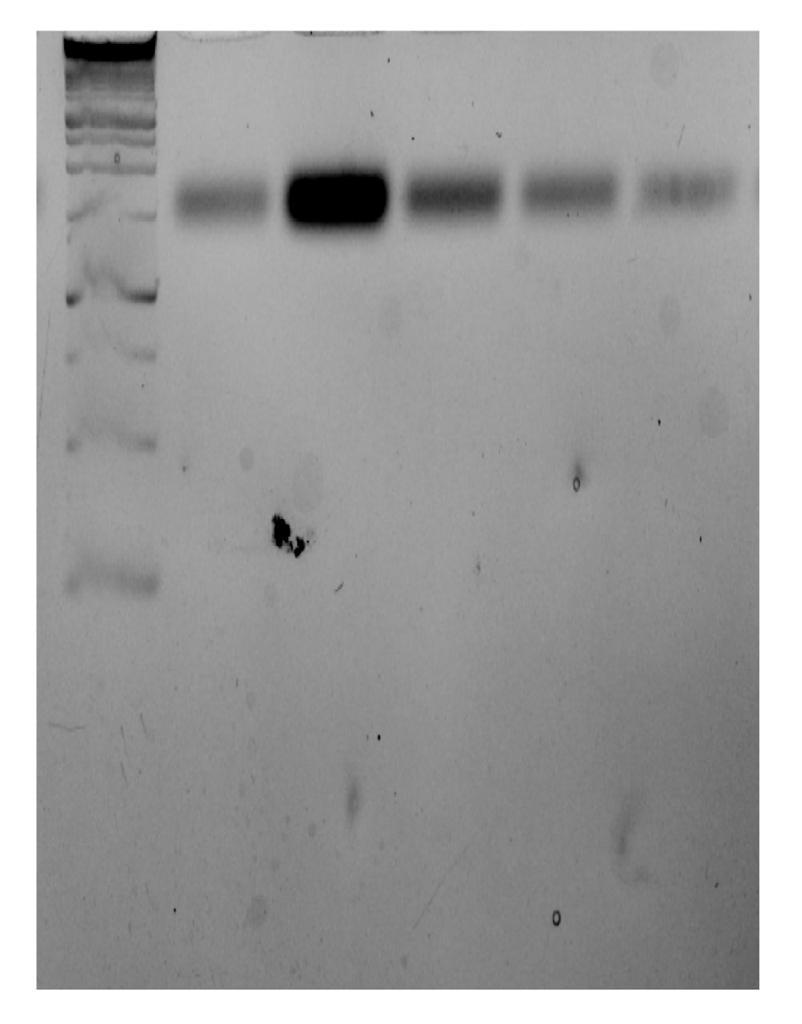

Supplement: Supplementary file 2 — Supplementary Information 2. [file 41598_2022_15551_MOESM2_ESM.jpg]

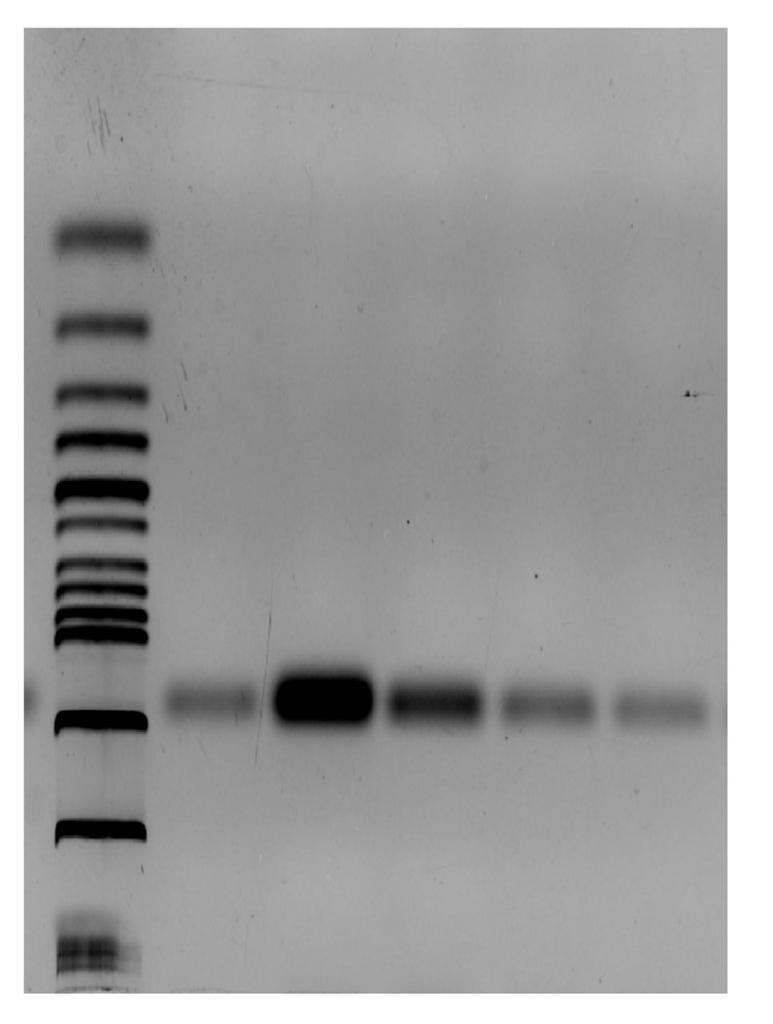

Supplement: Supplementary file 3 — Supplementary Information 3. [file 41598_2022_15551_MOESM3_ESM.jpg]

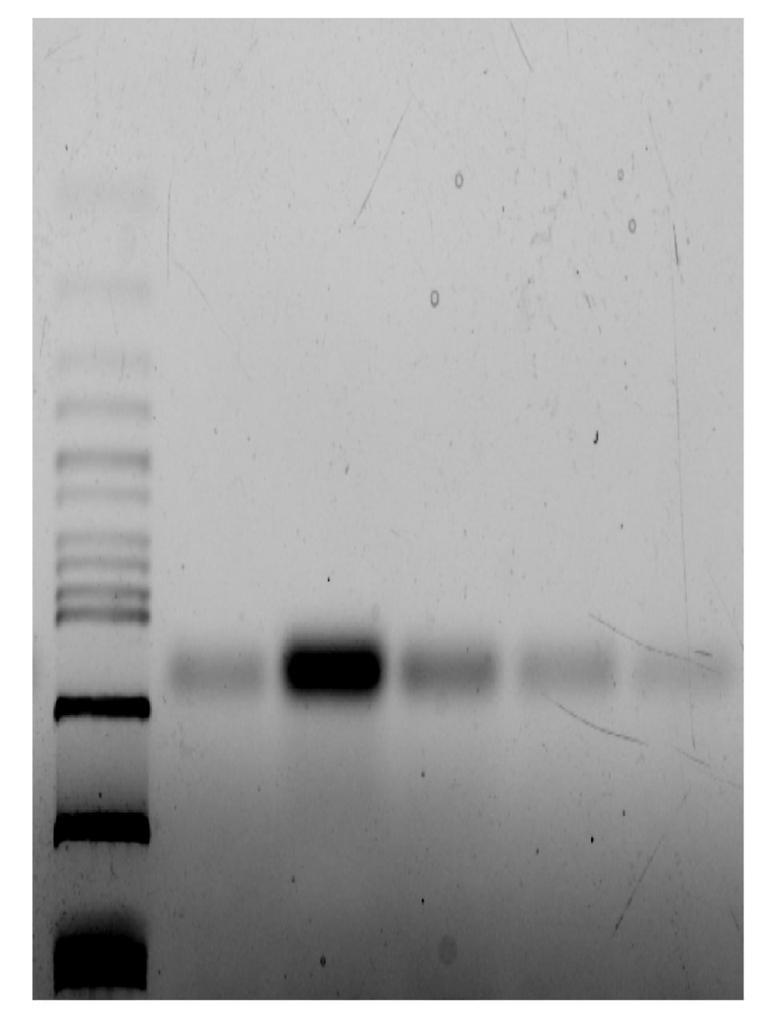

Supplement: Supplementary file 4 — Supplementary Information 4. [file 41598_2022_15551_MOESM4_ESM.jpg]

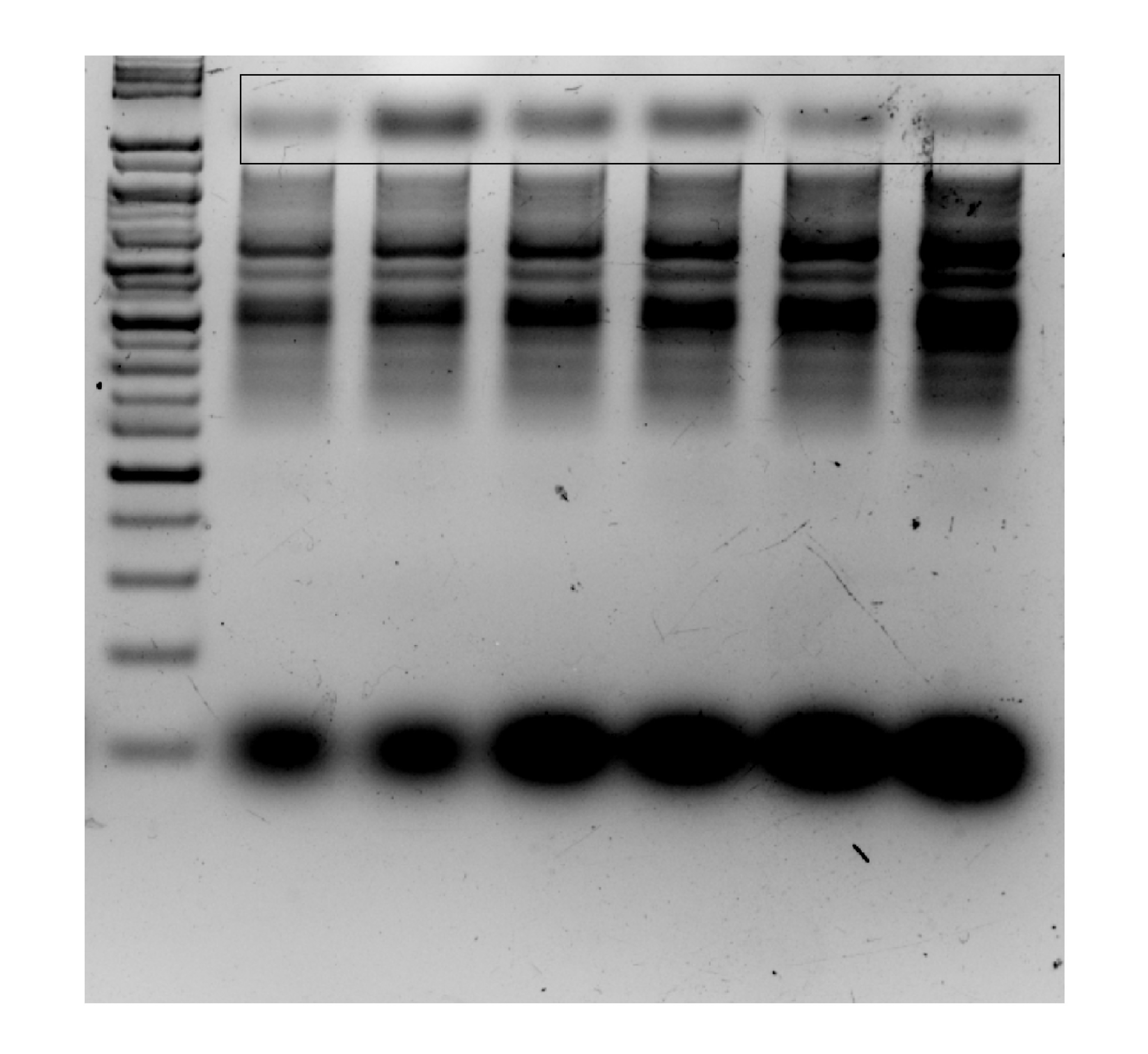

Supplement: Supplementary file 5 — Supplementary Information 5. [file 41598_2022_15551_MOESM5_ESM.jpg]

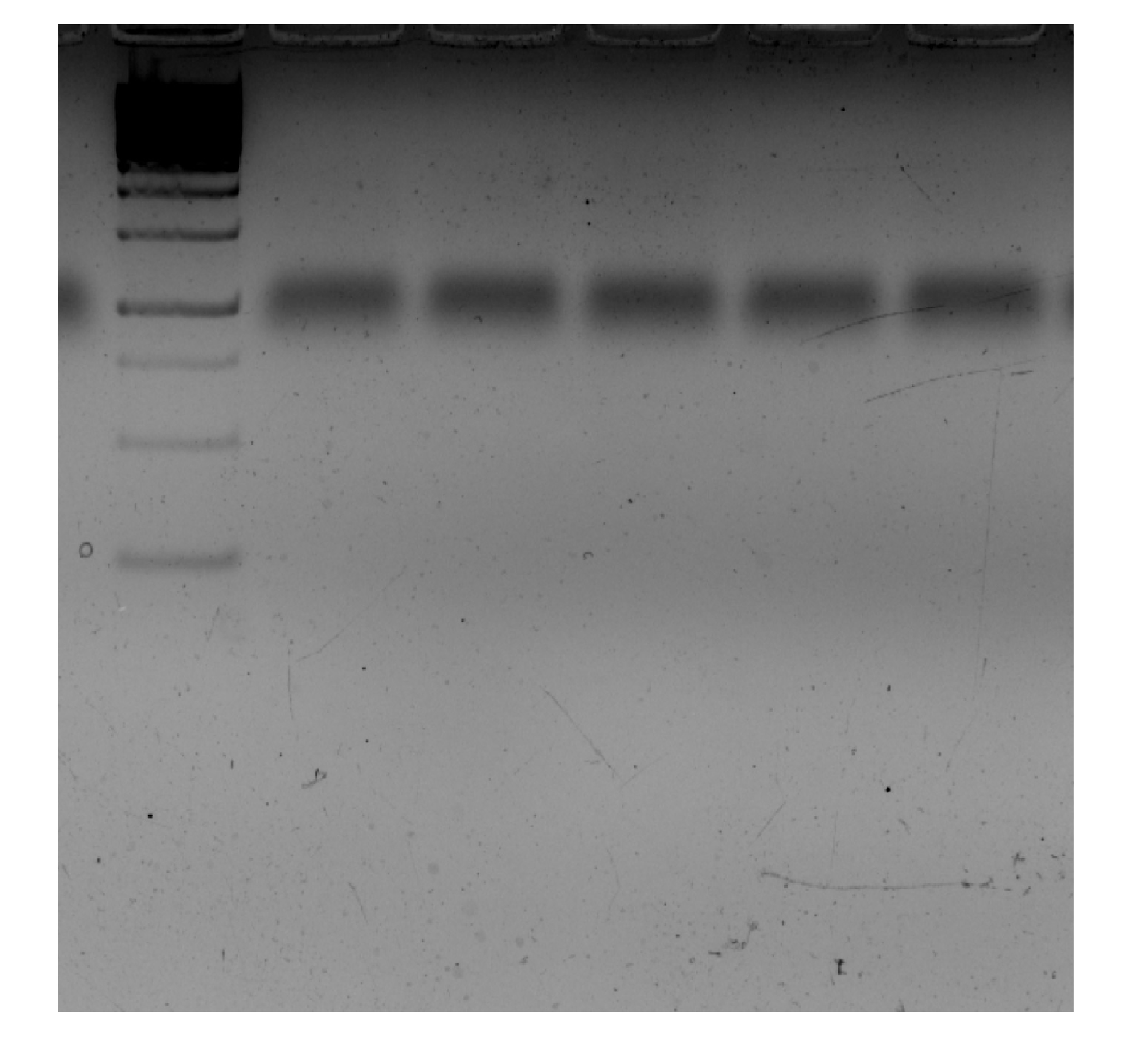

Supplement: Supplementary file 7 — Supplementary Information 7. [file 41598_2022_15551_MOESM7_ESM.jpg]

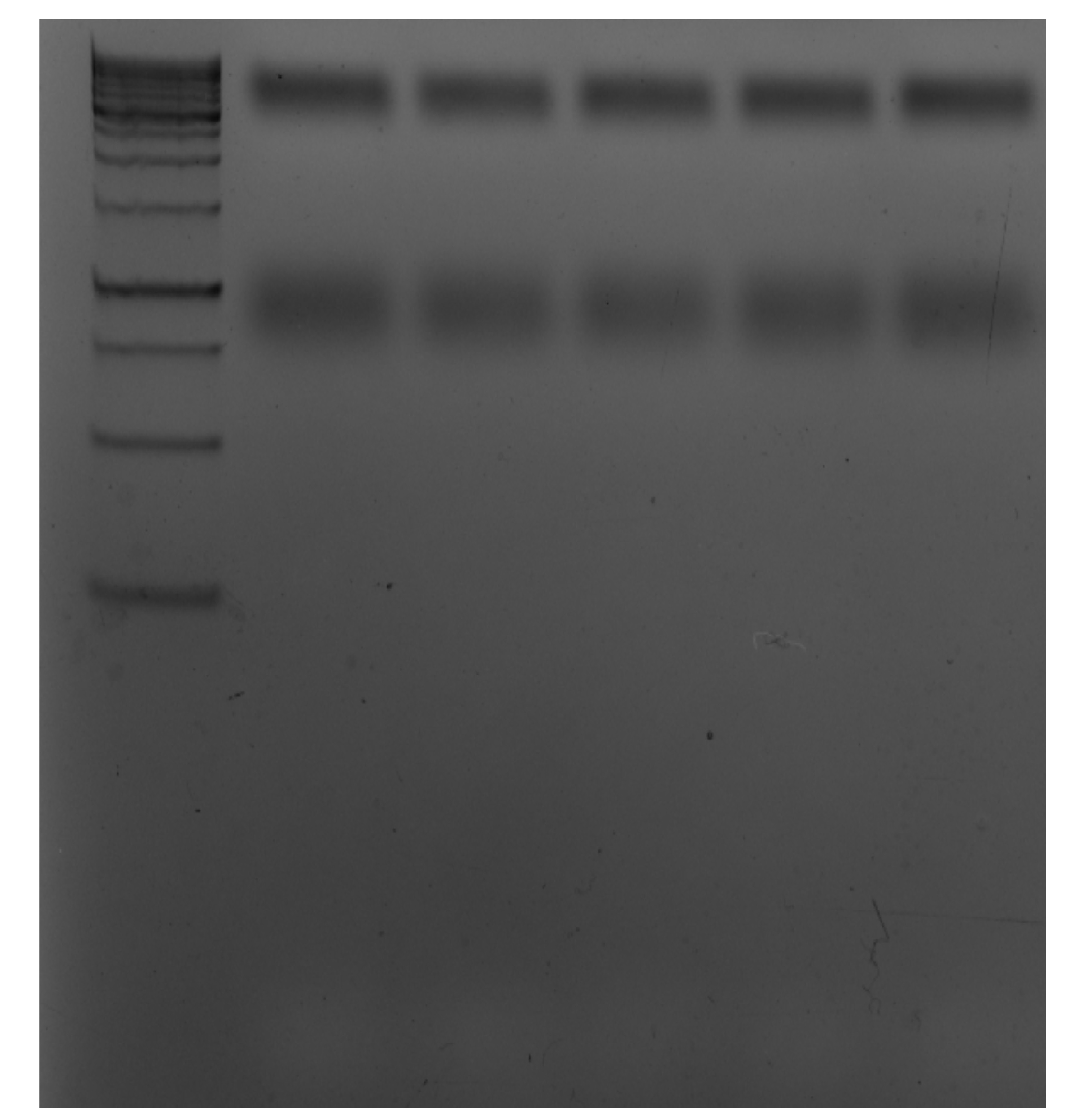

Supplement: Supplementary file 8 — Supplementary Information 8. [file 41598_2022_15551_MOESM8_ESM.jpg]

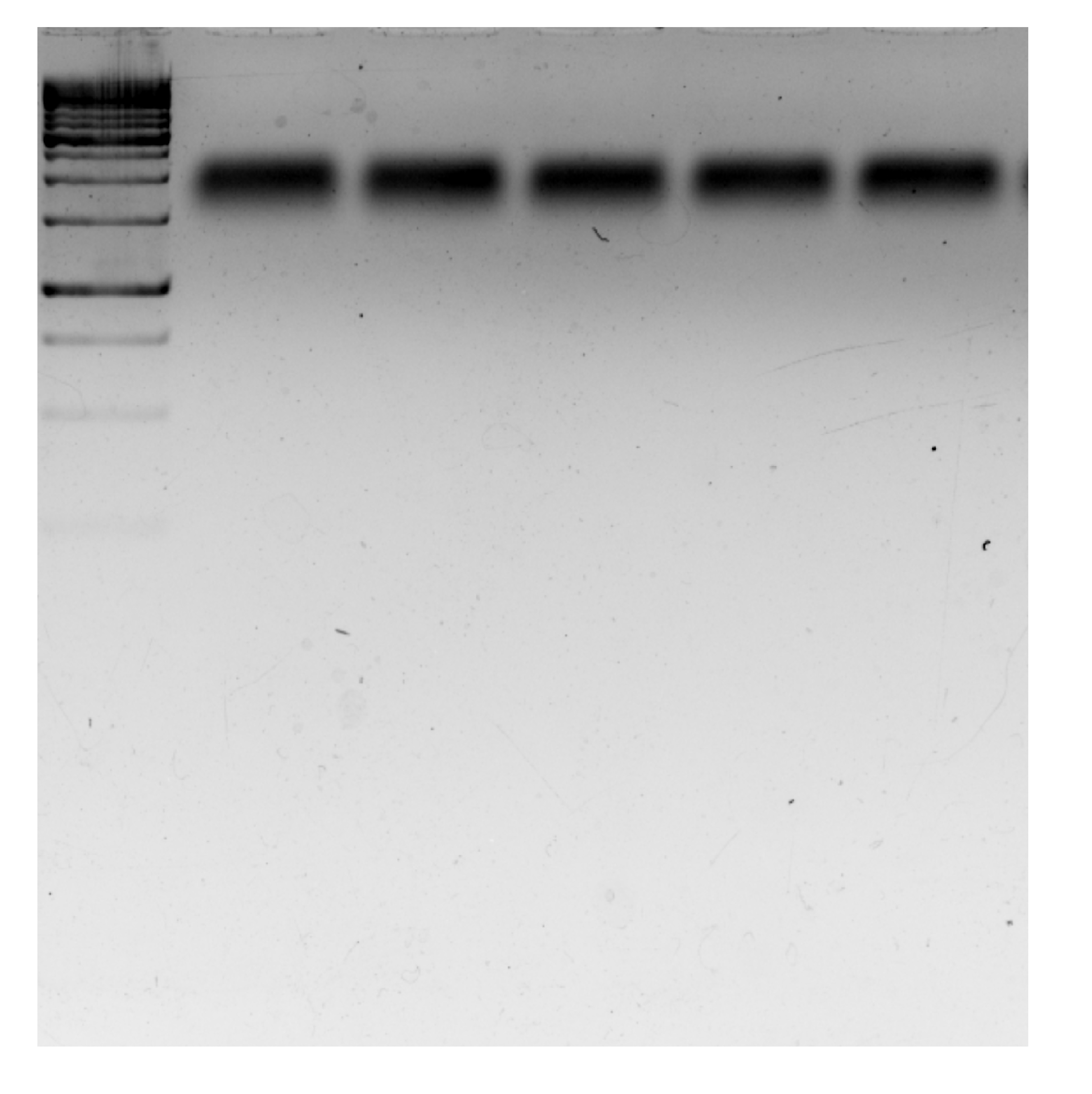

Supplement: Supplementary file 9 — Supplementary Information 9. [file 41598_2022_15551_MOESM9_ESM.jpg]
