# Supplementary material for: Anti-neoplastic action of Cimetidine/Vitamin C on histamine and the PI3K/AKT/mTOR pathway in Ehrlich breast cancer
Source: Sci Rep. 2022 Jul 7;12:11514. doi: 10.1038/s41598-022-15551-6 (PMC9262990; doi:10.1038/s41598-022-15551-6)
Supplement: Supplementary file 6 — Supplementary Information 6. [file 41598_2022_15551_MOESM6_ESM.pdf]

TNF

Normal control gp	Ehrlich gp	Vitamin C gp	Cimetidine gp	Cimetidine+Vitamin C gp
10.47	148.93	55.2	35.4	34.4
11.18	153.3	57.8	36.79	26.1
12.39	149	56.6	43.13	27.4
13.18	151.7	54.32	42.67	28.3
10.23	147.98	55.74	39	32.4
14.23	151.69	58.9	42.45	34
11.2	150	57.56	37.6	30.23
13.2	147.48	59.9	43.1	29.3

SOD

Normal control gp	Ehrlich gp	Vitamin C gp	Cimetidine gp	Cimetidine+Vitamin C gp
82	22.78	38.98	52	70.6
80	22.5	40.23	53.4	68.5
76	25.7	36.6	52.7	73.56
81.3	23.5	37.8	54.1	72.4
77	26	41	55.16	71.23
79	27.7	41.67	57.2	69.78
82	26.2	37.34	54.4	68
83	25.1	38.5	53.7	65.9

GSH

Normal control gp	Ehrlich gp	Vitamin C gp	Cimetidine gp	Cimetidine+Vitamin C gp
17.86	3.45	8.37	9.23	14
19.5	2.95	9.03	9.97	13.86
20.4	3.43	8.24	9.58	14.2
19.3	4.32	8.2	10.12	13.56
18.52	3.22	9.1	8.67	13.9
19	4	8.16	10.75	13.5
18.76	2.87	8.22	10.34	13.98
19	3.52	9.21	11.77	13.2

CEA

Normal control gp	Ehrlich gp	Vitamin C gp	Cimetidine gp	Cimetidine+Vitamin C gp
0.39	11.78	6	5.7	4.87
0.42	12.9	7.1	5.4	4.5
0.43	12.65	8.3	4.98	3.98
0.32	11.12	7.4	5.89	4.68
0.37	12.87	6.2	5.23	4.57
0.48	12.28	7.23	5.18	5.29
0.35	11.66	7	6.2	4.76
0.46	10.76	7.07	6.27	3.99

VEGF

Normal control gp	Ehrlich gp	Vitamin C gp	Cimetidine gp	Cimetidine+Vitamin C gp
45.1	153.45	87	67.7	53.12
48.03	147.34	83.5	66.8	50.31
47	149.6	82	68.2	55.89
49.5	148.2	84.69	76.8	52.45
53	152.67	81.68	71.23	52.2
52.4	154.6	85.3	67.35	55.36
53.23	152.28	89	68.46	57.9
51.8	149.9	86.9	73.51	53.96

MDA

Normal control gp	Ehrlich gp	Vitamin C gp	Cimetidine gp	Cimetidine+Vitamin C gp
18.87	87.5	59.5	45.42	36.67

19.23	86.8	56.98	42.28	29.54
20.31	90.6	55.7	46	32.8
18.9	89	59.83	44	27.84
22.1	97	61	40.07	29.4
21.2	88.27	55.4	46.5	29.87
15.6	89.04	61.65	47	34.9
24	92	62.2	49	35

CAMP

Normal control gp	Ehrlich gp	Vitamin C gp	Cimetidine gp	Cimetidine+Vitamin C gp
8.12	83.4	34.2	16	9.1
7.54	82.99	30.67	15.56	10.5
7.84	84.1	32.45	15.4	10.34
6.1	84.9	31.23	16.95	7.76
8.89	87.4	36.78	17.8	8.97
8.76	80.9	34.12	14.9	9.34
7.98	85.6	35.98	15.87	7.96
8.9	82.78	36.78	15.46	8.76

HISTAMINE

Normal control gp	Ehrlich gp	Vitamin C gp	Cimetidine gp	Cimetidine+Vitamin C gp
10.2	45	23.4	41.2	14.63
10.1	43.8	21.7	40.76	14.68
9.86	46.7	24.1	41	14.38
9.9	46.76	24	39.87	13.98
10.12	43.93	22.9	38.98	15.12
10.26	45.3	23.56	43.5	13.86
9.98	45.5	24.3	42.6	15.89
9.65	43.2	22.9	41.5	14.5

IRS-1

Normal control gp	Ehrlich gp	Vitamin C gp	Cimetidine gp	Cimetidine+Vitamin C gp
1	11	6	4.23	2.43
0.66	9.86	5.97	3.98	2.52
0.79	8.94	6.78	4.56	3.76
0.73	9.57	4.63	5.74	2.69
0.84	10.61	5.23	4.65	2.78
0.72	11.2	6.23	5.34	2.23
0.9	10	7.54	3.52	2.98
0.7	8.9	6.98	5.2	2.1

tumor volume (mm3)

	Ehrlich gp	Vitamin C gp	Cimetidine gp	Cimetidine+Vitamin C gp
0	0	0	0	0
9	126.13	122.1	121.98	123.09
11	400	227.98	140.29	125
13	600	429.65	160.5	126.5
15	1000	559.89	176.09	128.61
17	1550	597.32	210.45	129.75
19	1830	670.79	297.62	130.5
21	1961	716.5	335	132.7
23	2291.3	897	368	133.7
25	3068.3	1196.5	397	133.5

%Viability on MCF-7 cell line

	CONTROL	Vitamin C gp	Cimetidine gp	Cimetidine+Vitamin C gp
5.17	100	97.6	98.6	95
10.34	100	97.6	98.6	95

20.86	100	95	97.6	75
41.4	100	95	97.6	65
82.72	100	95	71	30
165.4	100	80	50	25
330.88	100	59	36.5	23
661.78	100	38	30	29
1323.52	100	35	17	20
2647.04	100	24	15	10
5294.08	100	17	14.1	10
10588.16	100	15	12	8
